# Meta-Analysis of Gene Expression and Identification of Biological Regulatory Mechanisms in Alzheimer's Disease

**DOI:** 10.3389/fnins.2019.00633

**Published:** 2019-07-03

**Authors:** Lining Su, Sufen Chen, Chenqing Zheng, Huiping Wei, Xiaoqing Song

**Affiliations:** ^1^Department of Basic Medicine, Hebei North University, Zhangjiakou, China; ^2^Institute of Educational Science, Zhangjiakou, China; ^3^Shenzhen RealOmics (Biotech) Co., Ltd., Shenzhen, China

**Keywords:** Alzheimer's disease, long non-coding RNA, microRNA, single nucleotide polymorphisms, network, meta-analysis

## Abstract

Alzheimer's disease (AD), also known as senile dementia, is a progressive neurodegenerative disease. The etiology and pathogenesis of AD have not yet been elucidated. We examined common differentially expressed genes (DEGs) from different AD tissue microarray datasets by meta-analysis and screened the AD-associated genes from the common DEGs using GCBI. Then we studied the gene expression network using the STRING database and identified the hub genes using Cytoscape. Furthermore, we analyzed the microRNAs (miRNAs), long non-coding RNAs (lncRNAs), and single nucleotide polymorphisms (SNPs) associated with the AD-associated genes, and then identified feed-forward loops. Finally, we performed SNP analysis of the AD-associated genes. Our results identified 207 common DEGs, of which 57 have previously been reported to be associated with AD. The common DEG expression network identified eight hub genes, all of which were previously known to be associated with AD. Further study of the regulatory miRNAs associated with the AD-associated genes and other genes specific to neurodegenerative diseases revealed 65 AD-associated miRNAs. Analysis of the miRNA associated transcription factor-miRNA-gene-gene associated TF (mTF-miRNA-gene-gTF) network around the AD-associated genes revealed 131 feed-forward loops (FFLs). Among them, one important FFL was found between the gene *SERPINA3*, hsa-miR-27a, and the transcription factor MYC. Furthermore, SNP analysis of the AD-associated genes identified 173 SNPs, and also found a role in AD for miRNAs specific to other neurodegenerative diseases, including hsa-miR-34c, hsa-miR-212, hsa-miR-34a, and hsa-miR-7. The regulatory network constructed in this study describes the mechanism of cell regulation in AD, in which miRNAs and lncRNAs can be considered AD regulatory factors.

## Introduction

Alzheimer's disease (AD) is the most well-reported neurodegenerative disease, and seriously affects patients' ability to perform daily activities. The characteristic pathological changes of AD are the formation of extracellular amyloid plaques by abnormal amyloid beta accumulation, the formation of intracellular neurofibrillary tangles by tau hyperphosphorylation, and neuronal loss with gliosis proliferation (Huttenrauch et al., 2018). The etiology and pathogenesis of AD have not yet been elucidated.

To identify the genetic variation in AD, large cohort studies have been carried out. The expression of stromal interaction molecule 1 (STIM1) protein decreases with the progression of neurodegeneration in AD by triggering voltage-regulated Ca^2+^ entry-dependent cell death (Pascual-Caro et al., 2018). The cerebrospinal fluid levels of C-X3-C motif chemokine ligand 1 which is a chemokine expressed by neurons, are decreased in AD dementia patients compared with controls (Perea et al., 2018). Genome-wide association studies (GWAS) studies have also revealed that some single nucleotide polymorphisms (SNPs) contribute to AD disease onset. These include common variants such as estrogen receptor 1 (*ESR1*), presenilin 1 (*PSEN1*), cholinergic receptor muscarinic 2 (*CHRM2*), cholinergic receptor muscarinic 3 (*CHRM3*), apolipoprotein E (*APOE*), apolipoprotein C1 (*APOC1*), and choline acetyltransferase (*CHAT*) (Zhou et al., 2014; Liu et al., 2016; Bagyinszky et al., 2018; Chee and Cumming, 2018; Li et al., 2018), and also rare variants in genes such as eukaryotic translation initiation factor 2 alpha kinase 3 (*EIF2AK3*) (Wong et al., 2018). EIF2AK3 is a single-pass type 1 membrane protein, which represses global protein synthesis as an endoplasmic reticulum stress sensor (Liu et al., 2012). Several SNPs within *EIF2AK3* appear to significantly increase the risk of AD (Liu et al., 2013), especially rs147458427, an SNP that changes arginine to histidine at amino acid 240 (R240H) (Wong et al., 2018). Although *EIF2AK3* polymorphisms are related to a risk of delayed AD (Liu et al., 2013), their function in neurodegenerative diseases is not very clear.

Different microRNAs (miRNAs) are also associated with the pathophysiology of several neurodegenerative diseases (Gaughwin et al., 2011; Zovoilis et al., 2011), including AD (Kumar and Reddy, 2018). miRNA-377 promotes cell proliferation and inhibits cell apoptosis by regulating the expression level of cadherin 13 (*CDH13*), thus participating in the development of AD (Liu et al., 2018). The level of miR-221 is downregulated in AD cases compared with controls, and it is potentially a new therapeutic target for increasing ADAM metallopeptidase domain 10 (ADAM10) levels in AD (Manzine et al., 2018).

Long non-coding RNAs (lncRNAs) are widely reported to be associated with various physiological and pathological processes, such as neurodegenerative diseases (Wang et al., 2016; Wang D. Q. et al., 2018). Brain cytoplasmic (BC) RNA is a lncRNA present at higher levels in the AD-affected region of the brain than in normal brain (Mus et al., 2007), and overexpression of BC in AD may cause synaptic/dendritic degeneratio (Wang H. et al., 2018).

miRNAs function by targeting mRNAs for cleavage or translational repression. lncRNAs may affect miRNA activity by chelating them, thereby upregulating the expression of the miRNA target genes. The study of gene regulatory networks is important for disease analysis (Rankin and Zorn, 2014). However, research on the association of these AD markers in the context of biological networks is limited. To understand AD correctly, regulatory networks involving genes, miRNAs, transcription factors (TF), and lncRNAs need to be studied.

## Materials and Methods

### Microarray Data Collection

We used “Alzheimer” as a keyword to search for gene expression studies from different brain tissues in the NCBI-GEO database (http://www.ncbi.nlm.nih.gov/geo/). Only original experimental studies that screened for genes differing between AD and healthy humans were selected. Our criteria were as follows: (1) the type of dataset was expression profiling by array; (2) the brain regions were the entorhinal cortex (EC), hippocampus (HIP), and medial temporal gyrus (MTG); (3) for each brain tissue dataset, the total number of available samples were ≥10. Finally, the samples from seven studies including three tissues (EC, HIP, and MTG) were screened out. We then performed a meta-analysis of three datasets from EC tissue (GSE48350, GSE5281, and GSE26927), five datasets from HIP tissue (GSE5281, GSE36980, GSE1297, GSE29378, and GSE48350), and two datasets from MTG tissue (GSE5281 and GSE84422). A detailed description of the microarray datasets is presented in Table 1. Detailed descriptions of the samples, including the brain regions, sex, and mean age, are provided in Table S1.

**Table 1:** Datasets used in the meta-analysis.

**Brain Regions**	**GEO accession**	**Sample size (AD/control)**	**Platform**	**PMID**
Entorhinal Cortex (EC)	GSE48350	AD = 15; HC = 39	GPL570: Affymetrix Human Genome U133 Plus 2.0 Array	23273601 (Berchtold et al., 2013)
	GSE5281	AD = 10; HC = 13	GPL570: Affymetrix Human Genome U133 Plus 2.0 Array	29937276 (Readhead et al., 2018)
	GSE26927	AD = 11; HC = 7	GPL6255: Illumina humanRef-8 v2.0 expression beadchip	25119539 (Durrenberger et al., 2015)
Hippocampus (HIP)	GSE5281	AD = 10; HC = 13	GPL570: Affymetrix Human Genome U133 Plus 2.0 Array	29937276 (Readhead et al., 2018)
	GSE36980	AD = 7; HC = 10	GPL6244: Affymetrix Human Gene 1.0 ST Array	23595620 (Hokama et al., 2014)
	GSE29378	AD = 31; HC = 32	GPL6947: Illumina HumanHT-12 V3.0 expression beadchip	23705665 (Miller et al., 2013)
	GSE48350	AD = 19; HC = 43	GPL570: Affymetrix Human Genome U133 Plus 2.0 Array	23273601 (Berchtold et al., 2013)
	GSE1297	AD = 22; HC = 9	GPL96: Affymetrix Human Genome U133A Array	14769913 (Blalock et al., 2004)
Medial temporal gyrus (MTG)	GSE5281	AD = 16; HC = 12	GPL570: Affymetrix Human Genome U133 Plus 2.0 Array	29937276 (Readhead et al., 2018)
	GSE84422	AD = 20; HC = 14	GPL96: Affymetrix Human Genome U133A Array	27799057 (Wang et al., 2016)

*AD, Alzheimer's disease; HC, healthy control*.

Searches were executed up to October 2017.

### Analysis of Individual Data

Background correction and normalization of each individual dataset were performed using Robust Multichip Averaging (RMA) (Taminau et al., 2012). The differentially expressed genes (DEGs) between AD and healthy control samples (HC) were computed using the limma package (Derkow et al., 2018) in R. Gene symbol probes without gene annotation were removed. When multiple probes were matched with the same gene, the average value was used as the expression value.

### Meta-Analysis of DEGs

**Figure 1:**
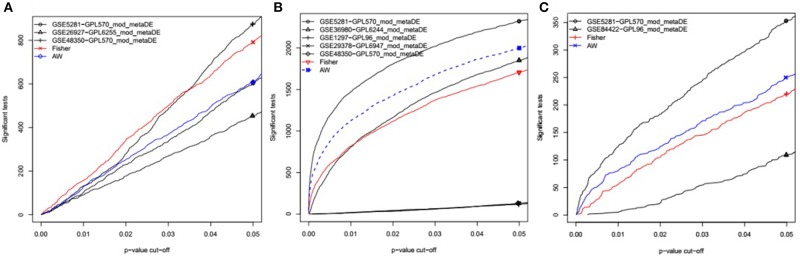
*P*-value vs. number of detected DEGs for individual analysis as well as meta-analysis. In each individual dataset, moderated-t statistics was used to generate p-values while adaptive weight and Fisher's methods were utilized to combine these *p*-values for meta-analysis. This figure is generated using the “MetaDE” package in R. **(A)** The datasets from Entorhinal Cortex; **(B)** the datasets from Hippocampus; **(C)** the datasets from Medial temporal gyrus.

Datasets from the same brain region (EC, HIP, or MTG) were combined to perform the meta-analysis. Initially, the data files were normalized using RMA. The normalized datasets were then merged using Fisher's exact test in the MetaDE package (Wang et al., 2012). The differentially expressed genes (DEGs) between AD and HC were selected using a *P* < 0.05 as the cut-off (Figure 1). In addition, the heterogeneity tests and differential expression analysis for each gene were analyzed using the ES algorithm of the MetaDE package in R (Wang et al., 2012). When multiple probes were matched with the same gene, we chose the average fold change of each probe. The thresholds of homogeneity were set as meta fold change > 1, tau^2^ = 0, and FDR > 0.05. The genes with tau^2^ = 0 and FDR > 0.05 were considered homogeneous and unbiased, from which the genes with a *P* < 0.05 in the Fisher's exact test of the MetaDE package were selected as DEGs. Tau^2^ represents the difference among study samples and reflects the heterogeneity between studies. The smaller the tau^2^ value, the smaller the heterogeneity.

In this study, sub-meta-analyses on males and females with GSEs from different tissues were performed. The methods of normalization, meta-analysis, heterogeneity detection of each gene and threshold for selecting DEGs were the same as above.

### RNA-Seq Data Analysis

We searched for gene expression studies from the NCBI-GEO database according to our criteria. The criteria were: (1) original studies between AD and healthy humans; (2) the type of dataset was expression profiling by high-throughput sequencing; (3) the brain regions used were EC, HIP, and MTG; (4) RNA-Seq data with poor quality controls were excluded. Finally, one gene expression dataset was selected, GSE67333, which uses samples from hippocampi brain regions and is based on GPL11154 platform information. Detailed information on these RNA-Seq samples is shown in Table S2. The available analyzed expression profiles of GSE67333 were used (Moradifard et al., 2018).

### Construction of the DEG PPI Network, and Identification and Further Analysis of the Hub Nodes

STRING is a protein interaction network analysis tool. The latest version of the STRING database is 11.0 (Szklarczyk et al., 2019), which covers more than 5,090 species and 24.6 million proteins and supports the upload of genome-level data sets. To determine which proteins encoded by the DEGs play a leading role in AD, the DEGs were subjected to STRING v.11.0 with medium confidence scores of 0.4. To identify the hub nodes, we visualized the protein–protein interaction (PPI) network using Cytoscape v.3.6.0 software and analyzed the topological properties of these nodes using the Network Analyzer tool based on the degree parameter (Shannon et al., 2003). Then we selected the nodes with high degrees and high closeness centrality values as hubs. The degree is the number of protein-specific interactions, and a high value reflects an important role in the network. The closeness centrality reflects the ability of nodes to influence other nodes in the network, which reveals the centrality of the node in the network.

### Identification of DEGs Associated With AD and Other Neurodegenerative Diseases

The Gene Radar online tool in GCBI (Shanghai, China, https://www.gcbi.com.cn/gclib/html/index) mainly uses the disease classification in the Mesh database to mine correspondence between genes and diseases from the PubMed database. We used Gene Radar to identify the DEGs associated with AD and those associated with other neurodegenerative diseases.

### Analysis of miRNAs Associated With the AD-Associated and Other Neurodegenerative Disease-Associated DEGs, and of lncRNAs Associated With These miRNAs

DIANA-Tarbase v.8.0, containing 670,000 unique experimentally-supported miRNA–gene pairs (Karagkouni et al., 2018), was used to analyze the miRNAs associated with the AD-associated and other neurodegenerative disease-associated DEGs. Then, the miRNAs associated with AD and other neurodegenerative diseases were filtered using the miRdSNP v.11.03 online database (Bruno et al., 2012).

DIANA-LncBase Experimental v.2, which provides more than 70,000 low- and high-throughput experimentally-supported miRNA–lncRNA interactions (Paraskevopoulou et al., 2016), was used to examine interactions between lncRNAs and these miRNAs. In our study, experimentally validated (prediction score ≥ 0.90) lncRNAs in human brain tissue were selected.

### Differentially Expressed miRNAs in AD by High-Throughput Data

High-throughput data on miRNAs in AD is rare, so we collected only one miRNA microarray dataset in GEO (GSE16759), which studied miRNA expression in AD patients and controls (Nunez-Iglesias et al., 2010). The differentially expressed miRNAs were screened using the GEO2R tool, which is an interactive web tool based on GEO query and limma R packages (Davis and Meltzer, 2007).

### Analysis of Transcription Factors Associated With the AD-Associated/Other Neurodegenerative Disease-Associated DEGs and AD-Associated/Other Neurodegenerative Disease-Associated miRNAs

To study the molecular regulatory mechanisms in AD, we built regulatory networks comprising AD-associated/other neurodegenerative disease-associated DEGs, TFs associated with these genes (gTFs), AD-associated/other neurodegenerative disease-associated miRNAs targeting these genes, and TFs related to these miRNAs (mTFs).

Information on the TF binding sites associated with these genes were studied using TRANSFAC (Fogel et al., 2005) based on the Match™ algorithm. The TRANSFAC database comprises eukaryotic transcription factors, DNA binding sites, and their effects on gene expression (Fogel et al., 2005). In our study, the matrix similarity score (MSS) and the core similarity score (CSS) were used to estimate the result. The threshold values of MSS and CSS for selection were both score = 1.

Regulatory information on the TFs associated with these miRNAs was analyzed using TransmiR v.2.0 database, an updated TF–miRNA regulation database (Wang et al., 2010). In our study, the literature-curated TF–miRNA regulations and the TF–miRNA interactions from ChIP-Seq evidence in human neural tissue were selected.

### Verification of FFL Between the Gene SERPINA3, hsa-miR-27a and TF MYC

In order to verify the positive finding, FFL between the gene SERPINA3, hsa-miR-27a, and TF MYC, we collected GSE16759 dataset, which jointly profiled mRNA and miRNA expression in AD patients and controls (Nunez-Iglesias et al., 2010). The differentially expressed mRNA and miRNAs were screened using the GEO2R tool (Davis and Meltzer, 2007).

To further verify the positive finding, GSE46579 dataset which studied miRNA expression in AD patients and controls blood and GSE97760 dataset which studied mRNA expression in AD patients and controls blood were collected. The available analyzed expression profiles of GSE46579 were used (Leidinger et al., 2013). The differentially expressed mRNA were also screened using the GEO2R tool.

### SNP Analysis of the AD-Associated DEGs

To identify the AD-associated SNPs, SNP analysis of the AD-associated DEGs was performed. We used miRdSNP v.11.03 (Bruno et al., 2012) and LincSNP v.2.0 (Ning et al., 2014) to identify AD-associated SNPs associated with AD-associated DEGs (Yousef, 2015) Chromosome locus and allele gene information associated with each of the SNPs were received from dbSNP database (https://www.ncbi.nlm.nih.gov/snp/?term=).

## Results

### Analysis of Individual Datasets

**Figure 2:**
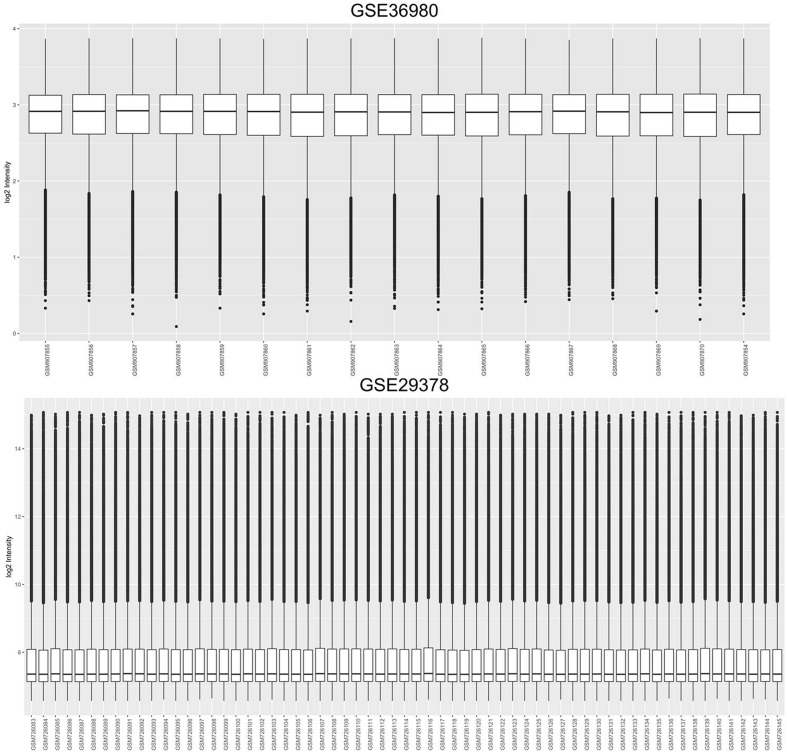
Cassette figures of the expression data after standardization.

Each individual dataset selected for use in the meta-analysis were corrected and normalized using the oligopackage (Liu et al., 2018) in R. All results are shown in Additional Figures, parts of which are shown in Figure 2.

### Meta-Analysis of DEGs

To identify common DEGs in different brain regions between AD and healthy controls, microarray datasets (Table 1) from three different brain regions (EC, HIP, and MTG) were meta-analyzed using the “MetaDE” package in R. With the threshold of *P* < 0.05 in Fisher's exact test, 781 DEGs in the EC brain region, 1707 DEGs in the HIP brain region and 220 DEGs in the MTG brain region were obtained. Then, with the homogeneity thresholds of meta fold change > 1, tau^2^ = 0, and FDR > 0.05, the DEGs with *P* < 0.05 in the meta-analysis from each tissue were collected and are shown in Table 2. The final results identified 183 DEGs in the EC brain region (120 upregulated and 63 downregulated), nine DEGs in the HIP brain region (four upregulated and five downregulated) and 15 DEGs in the MTG brain region (14 upregulated and one downregulated). A total of 207 DEGs were identified between the AD and HC samples (Table 2). More details about the DEGs are presented in Table 3, including the meta-expression between AD and HC and the Qpval in the meta-analysis. These 207 genes were identified as DEGs for the subsequent analysis.

**Table 2:** DEGs of Alzheimer's disease in different brain regions.

**Brain partition**	**Up**	**Down**
Entorhinal Cortex (EC)	120	63
Hippocampus (HIP)	4	5
Medial temporal gyrus (MTG)	14	1

**Table 3:** Diferentially expressed genes (DEGs) identifed in the meta-analysis of AD datasets.

**Brain tissue**	**Gene symbols**	**Meta. fold change**	**Meta. FDR**	**Meta. z-score**	**Brain tissue**	**Gene symbols**	**Meta. fold change**	**Meta. FDR**	**Meta. z-score**
**Down regulated**					**UP regulated**				
Entorhinal Cortex (EC)	*ABHD8*	−0.695534942	0.637337464	0.000948744	Entorhinal Cortex (EC)	*ENPP2*	0.44049447	0.429551194	0.033759913
	*ACTR1A*	−0.527310094	0.918943259	0.01124593		*FAM133B*	0.712276458	0.920723021	0.000694549
	*ADAP1*	−0.826978871	0.876417161	8.7236E-05		*FAM189A2*	0.489157939	0.410574925	0.018954003
	*ALDOA*	−0.384145613	0.425112103	0.062660907		*FANCL*	0.470511307	0.953386722	0.023061608
	*ATP13A2*	−0.762035644	0.766539678	0.000296769		*FBXO15*	0.993507129	0.757129441	3.75657E-06
	*ATP5D*	−0.723661334	0.534708953	0.000580599		*FUT9*	0.780769619	0.873935779	0.000212873
	*BRSK2*	−0.533706412	0.999573802	0.010248351		*GFAP*	0.398018735	0.376701655	0.054426496
	*BSG*	−0.516925038	0.413512963	0.013242341		*GTF2H5*	0.657325258	0.789398314	0.001738459
	*C1QTNF4*	−0.457508928	0.431360385	0.027673846		*HERC5*	0.574139491	0.947265969	0.005903665
	*C9ORF16*	−0.604569827	0.566387182	0.00393522		*HLA-A*	0.373842817	0.58003196	0.069323817
	*CA11*	−0.583952921	0.379126602	0.005356457		*HLA-DMA*	0.513914113	0.587127773	0.013644294
	*CCDC3*	−0.53490691	0.450261271	0.010464563		*HLA-DPA1*	0.353794692	0.657898571	0.084630186
	*CHGA*	−0.511634157	0.706121833	0.013873028		*HSPB8*	0.464862559	0.725668737	0.024925703
	*COX7B*	−0.633058899	0.522542766	0.002608732		*ID3*	0.556454024	0.75946288	0.007598297
	*CPLX1*	−0.491697637	0.654796636	0.017956424		*IFI16*	0.515223575	0.589948306	0.013365473
	*DMTN*	−0.517050939	0.54557447	0.013023207		*IFT80*	0.621059722	0.863851754	0.00303072
	*DNM1*	−0.748921362	0.851616972	0.000365222		*IGSF6*	0.565723712	0.935253446	0.006629518
	*EDF1*	−0.590415465	0.79983381	0.004719509		*IQCK*	0.824410953	0.785004124	9.22448E−05
	*EIF5A*	−0.561832967	0.763720591	0.007062777		*IRF8*	0.666513932	0.459048511	0.001602805
	*EPHB6*	−0.473978863	0.925286791	0.022141665		*KAT2B*	0.43895503	0.572883821	0.034051256
	*FAIM2*	−0.39169712	0.423434914	0.057746056		*KCTD12*	0.448754813	0.422874061	0.030866516
	*FXYD7*	−0.603890632	0.92041114	0.003888889		*KMT5B*	0.722220565	0.565274027	0.000604808
	*GABRA1*	−0.446962428	0.938560944	0.030608148		*LAP3*	0.600061529	0.752107238	0.004131397
	*GAPDH*	−0.543132906	0.903040583	0.009132231		*LBR*	0.545302628	0.633986877	0.009041239
	*GNA11*	−0.577416641	0.843517364	0.005654896		*LIX1*	0.5214595	0.717112961	0.012268136
	*GNAS*	−0.603746148	0.849586058	0.003904333		*LPAR4*	0.921307459	0.681435858	1.21045E-05
	*GNB5*	−0.411630184	0.446016846	0.046627431		*MAFB*	0.645061604	0.567530214	0.002210118
	*GNG3*	−0.511352374	0.60511663	0.013986977		*MAN2A1*	0.430556072	0.512746474	0.037462643
	*HCFC1R1*	−0.779144243	0.959187083	0.000221638		*MEGF10*	0.469347495	0.468585217	0.023860088
	*HSPBP1*	−0.526128712	0.837799088	0.011457968		*MYBPC1*	0.502311812	0.579998504	0.015894899
	*INA*	−0.386288506	0.407714233	0.061235913		*N4BP2L2*	0.898485485	0.893181433	1.66959E-05
	*IPCEF1*	−0.427095032	0.568091293	0.039036647		*NFASC*	0.519910402	0.47159205	0.012649219
	*KCNH3*	−0.689163117	0.88872707	0.001033475		*NPL*	0.585010189	0.930952647	0.005073462
	*KCNS1*	−0.420524054	0.753519306	0.041720093		*NQO1*	0.583983538	0.877051724	0.005170715
	*L1CAM*	−0.537297073	0.853172878	0.00989732		*NUP133*	0.628705637	0.535452706	0.002834544
	*MAP1A*	−0.665635022	0.662053461	0.001574005		*OGFRL1*	0.73737742	0.988179105	0.000443276
	*MIF*	−0.726002013	0.612623491	0.000561817		*P2RY1*	0.615041285	0.619500158	0.003352534
	*MINK1*	−0.41901789	0.512203346	0.042751064		*PALLD*	0.552681973	0.729315709	0.008066199
	*MLF2*	−0.631968567	0.729765354	0.002633776		*PCMTD2*	0.722905062	0.996225613	0.000571834
	*MLST8*	−0.808675599	0.408001512	0.000135237		*PIGF*	0.714748936	0.831142721	0.00067827
	*NARS*	−0.536469861	0.622164429	0.010066784		*PLEK*	0.631049594	0.92097513	0.002621254
	*NDUFV3*	−0.750515002	0.962465905	0.000351448		*PLSCR4*	0.499091341	0.456919204	0.01664371
	*NPDC1*	−0.560193708	0.812610721	0.007166708		*PLXDC2*	0.563414288	0.610862181	0.006953418
	*NPM2*	−0.692171755	0.892705473	0.000990483		*PPM1K*	0.663355287	0.794773659	0.001602805
	*NRGN*	−0.543671699	0.759036634	0.009171049		*PRDX1*	0.486533018	0.875508707	0.018985308
	*NRSN2*	−0.689295698	0.797483245	0.001049336		*PRDX6*	0.528459065	0.898873556	0.011077302
	*NRXN2*	−0.658068088	0.648530052	0.001734285		*PRPF38B*	0.717412208	0.47672117	0.000668253
	*OTUB1*	−0.510602162	0.838921288	0.013990734		*PTPN13*	0.740120096	0.847406651	0.000426997
	*PCSK1N*	−0.577285436	0.708629247	0.005726271		*PTTG1IP*	0.528946561	0.592564809	0.01120586
	*POLR2I*	−0.447268575	0.723136773	0.030829368		*QKI*	0.482455410	0.56935108	0.020327239
	*PPFIA3*	−0.588045143	0.568361574	0.004924869		*RAB10*	0.571514428	0.96471241	0.006130729
	*RAD23A*	−0.706497339	0.503752324	0.000808498		*RB1*	0.771349913	0.70327628	0.000265047
	*RPH3A*	−0.603970489	0.831838989	0.003941481		*RBL2*	0.596654578	0.955853017	0.004295016
	*SEZ6L2*	−0.760423859	0.845850174	0.000308039		*RHOBTB3*	0.517224591	0.635022215	0.012995242
	*SLC17A6*	−0.533301618	0.430952335	0.010548042		*RNF19A*	0.592985615	0.811236679	0.004594707
	*SLC25A6*	−0.655147169	0.610182759	0.001839469		*SERPINA3*	0.260148507	0.410252167	0.199003256
	*SYN1*	−0.500531256	0.401736659	0.01638868		*SFRP2*	0.710029305	0.949693908	0.000717923
	*TMSB10*	−0.532612324	0.697663977	0.010507137		*SLC16A9*	0.382811918	0.378195934	0.06339803
	*TNPO2*	−0.614679785	0.930625382	0.003314968		*SLC44A1*	0.638101475	0.762520335	0.002367894
	*TOMM40*	−0.738938287	0.985360607	0.000431171		*SLC47A2*	0.453485364	0.582897757	0.028596711
	*TUBB4A*	−0.489135484	0.590668267	0.018714834		*SMAD5*	0.727328341	0.605345958	0.000553469
	*TUBB4B*	−0.555933702	0.581279394	0.007710577		*SMC3*	0.704413915	0.748404935	0.000804324
	*USP11*	−0.458379228	0.748899819	0.026995158		*SMG1*	0.753030042	0.746878908	0.000341013
Hippocampus (HIP)	*RPS27A*	−0.506389968	0.498368501	0.000441679		*SOX9*	0.426447386	0.834971993	0.038857167
	*TPD52L2*	−0.495625518	0.513661726	0.000568354		*SPARC*	0.434472551	0.909292556	0.035391101
	*IFI27*	−0.457966173	0.686297499	0.001275153		*SPATA13*	0.71020661	0.728056464	0.000732949
	*HBB*	−0.386701035	0.420669794	0.005961549		*SPP1*	0.493881628	0.452317782	0.017825778
	*NCAN*	−0.237122792	0.418570953	0.082322179		*SRSF6*	0.458207354	0.831801769	0.026846982
Medial temporal gyrus(MTG)	*BAIAP3*	−0.653066361	0.435361527	0.006816604		*STARD7*	0.494066945	0.774264489	0.017368311
						*STOM*	0.454651040	0.432990835	0.028810836
Up regulated						*SUMF1*	0.745609227	0.904439144	0.000385675
Entorhinal Cortex (EC)	*ACADM*	0.509396446	0.472281771	0.014636865		*TAC1*	0.391075996	0.474031506	0.057949745
	*ACTL6A*	0.63989583	0.918366926	0.002292345		*TJP2*	0.532971400	0.750652089	0.010483346
	*ADAMTSL3*	0.55218035	0.526447536	0.008121713		*TMEM123*	0.62618571	0.657410829	0.002886301
	*ADAP2*	0.58201966	0.741648989	0.00536564		*TPD52L1*	0.489260339	0.506535512	0.018809583
	*AK3*	0.624295669	0.436260102	0.003014442		*TPT1*	0.300133425	0.368223777	0.141755572
	*AKR1C3*	0.610232089	0.96965544	0.003533684		*TRIM22*	0.678534839	0.832321099	0.001261374
	*ALDH9A1*	0.632866364	0.75439915	0.002612489		*TRMT13*	0.793756883	0.369197713	0.000174472
	*AMOTL2*	0.525992143	0.439842541	0.011663327		*TSPAN6*	0.629323862	0.934431325	0.002691794
	*ANGPT1*	0.541370626	0.948471299	0.009286251		*UNC50*	0.538042189	0.675647763	0.009896486
	*ANXA5*	0.519090679	0.491664199	0.012875449		*VPS13C*	0.575598517	0.383372507	0.006035562
	*APBB1IP*	0.608077987	0.953355887	0.003667668		*WDR11*	0.838741219	0.890945758	6.72009E-05
	*APLNR*	0.370607123	0.853230372	0.070987979		*WRN*	0.697603632	0.573479036	0.0009141
	*ARFGAP3*	0.701645153	0.629505964	0.000882795		*ZFAND6*	0.669587769	0.788327401	0.001442107
	*ARRDC4*	0.537624139	0.502111086	0.00999833		*ZNF536*	0.408408649	0.397147119	0.048474831
	*ATG4C*	0.624765761	0.440666196	0.002969363		*ZNF770*	0.875598858	0.482428475	3.13048E-05
	*ATRAID*	0.601603100	0.775419452	0.004065448	Hippocampus (HIP)	*PCSK1N*	0.225974152	0.416500814	0.09711283
	*B2M*	0.353023046	0.579830231	0.085440354		*GJA1*	0.252228568	0.674305099	0.035832633
	*BBOX1*	0.644908898	0.465640403	0.002237249		*MT1M*	0.289185091	0.603279837	0.035832633
	*BMI1*	0.542056551	0.673043701	0.00935053		*PLSCR4*	0.29691696	0.406351363	0.031675355
	*C3*	0.421657492	0.839037764	0.040981301	Medial temporal gyrus(MTG)	*AEBP1*	0.70464363	0.521628759	0.003562337
	*C5ORF15*	0.804720546	0.741920597	0.000134819		*AFFX-HUMRGE/M10098_3_AT*	0.368444288	0.511222692	0.120022659
	*CAPN2*	0.535982721	0.760836799	0.010108106		*AFFX-HUMRGE/M10098_5_AT*	0.550884703	0.476493887	0.021509016
	*CAPS*	0.483265112	0.560111705	0.019921529		*AFFX-HUMRGE/M10098_M_AT*	0.467365996	0.623774962	0.050219844
	*CD81*	0.51520138	0.871213337	0.013160531		*COX17*	0.818729013	0.427171437	0.000795487
	*CEBPB*	0.380312809	0.378471552	0.065611904		*EEF2*	0.386796708	0.358680034	0.103799055
	*CLCA4*	0.65055172	0.434917261	0.002025628		*GOT2*	0.468316719	0.34099237	0.050357246
	*CLU*	0.447778971	0.448785848	0.030793889		*HBA1*	0.715632134	0.485832639	0.003132774
	*CMTR2*	0.629566266	0.552935904	0.00274856		*HSPB1*	0.470339479	0.624218434	0.048780735
	*COMMD3*	0.534472888	0.71040471	0.010343935		*MT3*	0.469118942	0.458310927	0.049672645
	*CP*	0.523351329	0.715531248	0.011868269		*NME2*	0.647538352	0.353484653	0.007327162
	*CSRP2*	0.533389066	0.653399315	0.010396527		*SEPP1*	0.517802828	0.438589877	0.03046524
	*DDIT4L*	0.461914904	0.984152235	0.025550129		*SEPW1*	0.477043287	0.45908091	0.045928551
	*DOCK4*	0.684322338	0.516002378	0.001146172		*ZBTB16*	0.515674996	0.345619847	0.031356668
	*DYNLT1*	0.614852884	0.938093709	0.003331664					

*The DEGs were listed based on homogeneity detection FDR and meta-analysis fold change. AD, Alzheimer's disease; HC, healthy control*.

**Figure 3:**
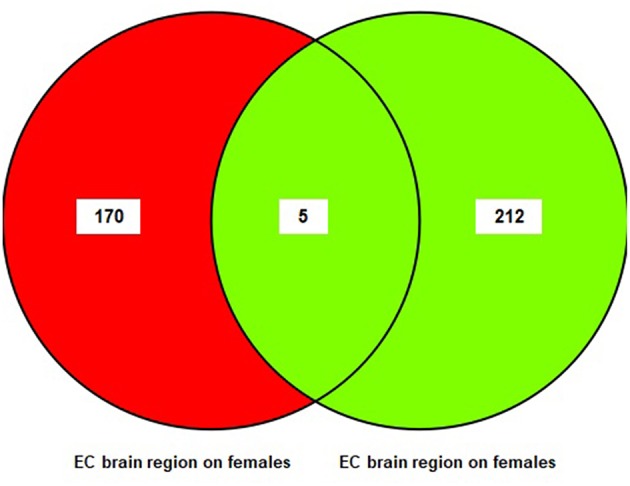
The number of common genes obtained from entorhinal Cortex (EC) in females and males visualized by a Venn diagram.

A sub-meta-analysis on females and males was performed using datasets from each brain region (EC, HIP, and MTG). The results of the sub-meta-analysis of the EC brain region in males and females revealed that there were 217 DEGs in males (176 upregulated and 41 downregulated), of which 212 were male-specific, and 175 DEGs in females (five upregulated and 170 downregulated), of which 170 were female-specific (Table S3). Only five common DEGs were identified in females and males. The result of this analysis is also shown in a Venn diagram (Figure 3).

The results of the sub-meta-analysis of the HIP brain region in males and females showed that there were 11 DEGs in males (one upregulated and 10 downregulated), and 28 DEGs in females (10 upregulated and 18 downregulated). No overlapping DEGs were obtained between females and males (Table S4).

**Figure 4:**
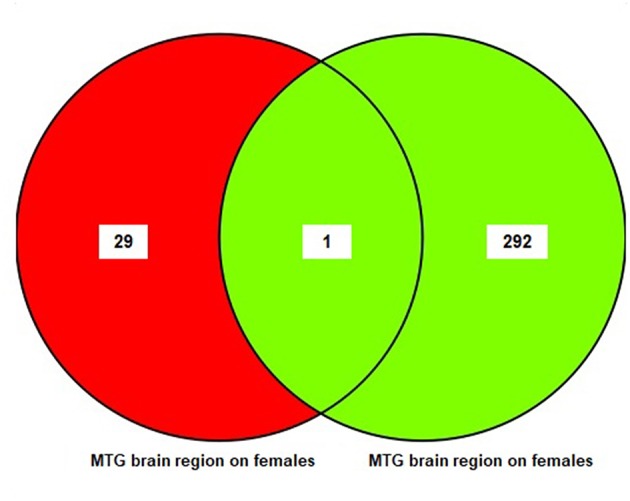
The number of common genes obtained from medial temporal gyrus (MTG) in females and males visualized by a Venn diagram.

The results of the sub-meta-analysis of the MTG brain region in males and females showed that there were 293 DEGs in males (11 upregulated and 282 downregulated), and 30 DEGs in females (19 upregulated and 11 downregulated) (Table S5). Only one common DEG was identified in females and males. The result of this analysis is also shown in a Venn diagram (Figure 4).

### Analysis of Gene Expression in AD by RNA Sequencing

To further validate the results from the microarray data with public RNA-Seq data, we selected datasets that used the same brain regions as the microarray datasets. Hence, the DEGs between AD and HC were analyzed in the GSE67333 RNA-Seq dataset. This revealed that 1102 DEGs were filtered from GSE67333 with a threshold of *P* < 0.05 and fold change ≥ 1.23 (Moradifard et al., 2018). Detailed information on these DEGs is shown in Table S6. Then, we analyzed the common DEGs between the RNA-Seq and microarray data, which revealed 72 common DEGs in the HIP brain region in both the RNA-Seq and microarray data.

### Identification of AD-Associated or Other Neurodegenerative Disease-Associated DEGs

AD-associated and other neurodegenerative disease-associated genes were identified from the DEGs using the Gene Radar tool from the GCBI online software. Out of the 207 total DEGs, 57 had previously been shown to be associated with AD (AD-associated genes; 34 upregulated and 23 downregulated), and 43 DEGs had previously been shown to be associated with several other neurodegenerative diseases (other neurodegenerative disease-associated genes; 30 upregulated and 13 downregulated), such as multiple sclerosis, Parkinson's disease (PD) and Huntington disease (HD) (Table 4). Overall, the number of upregulated genes was greater than the number of downregulated genes. These AD-associated or other neurodegenerative disease-associated DEGs are important genes for further research. The detailed down/upregulated status of the 57 AD-associated DEGs in each individual dataset is provided in Table 5.

**Table 4:** AD and other neurodegenerative diseases associate genes identified from DEGs using GCBI online software.

**Expressionin meta-analysis**	**Gene symbol**	**Brain partition**	**Gene name**	**Corresponding neurodegenerative disease**
Down-regulated	*ADAP1*	EC	ArfGAP with dual PH domains 1	AD
	*MLST8*	EC	MTOR associated protein, LST8 homolog	AD
	*HCFC1R1*	EC	Host cell factor C1 regulator 1	AD
	*NDUFV3*	EC	NADH:ubiquinone oxidoreductase subunit V3	AD
	*DNM1*	EC	Dynamin 1	AD
	*TOMM40*	EC	Translocase of outer mitochondrial membrane 40	AD
	*RAD23A*	EC	RAD23 homolog A, nucleotide excision repair protein	AD
	*RPH3A*	EC	Rabphilin 3A	AD
	*FXYD7*	EC	FXYD domain containing ion transport regulator 7	AD
	*GNAS*	EC	GNAS complex locus	AD
	*PCSK1N*	EC	Proprotein convertase subtilisin/kexin type 1 inhibitor	AD
	*TUBB4B*	EC	Tubulin beta 4B class IVb	AD
	*NRGN*	EC	Neurogranin	AD
	*GAPDH*	EC	Glyceraldehyde-3-phosphate dehydrogenase	AD
	*L1CAM*	EC	L1 cell adhesion molecule	AD
	*BRSK2*	EC	BR serine/threonine kinase 2	AD
	*SLC17A6*	EC	Solute carrier family 17 member 6	AD
	*HSPBP1*	EC	HSPA (Hsp70) binding protein 1	AD
	*DMTN*	EC	Dematin actin binding protein	AD
	*SYN1*	EC	Synapsin I	AD
	*GNB5*	EC	G protein subunit beta 5	AD
	*INA*	EC	Internexin neuronal intermediate filament protein alpha	AD
	*RPS27A*	HIP	Ribosomal protein S27a	AD
	*ATP13A2*	EC	ATPase 13A2	PD
	*MAP1A*	EC	Microtubule associated protein 1A	PD
	*SLC25A6*	EC	Solute carrier family 25 member 6	PD
	*ATP5D*	EC	ATP synthase, H+ transporting, mitochondrial F1 complex, delta subunit	Multiple sclerosis
	*MLF2*	EC	Myeloid leukemia factor 2	Multiple sclerosis
	*CA11*	EC	Carbonic anhydrase 11	Multiple sclerosis
	*ACTR1A*	EC	ARP1 actin-related protein 1 homolog A, centractin alpha	Multiple sclerosis
	*OTUB1*	EC	OTU deubiquitinase, ubiquitin aldehyde binding 1	Multiple sclerosis
	*EPHB6*	EC	EPH receptor B6	Multiple sclerosis
	*C1QTNF4*	EC	C1q and tumor necrosis factor related protein 4	Multiple sclerosis
	*ALDOA*	EC	Aldolase, fructose-bisphosphate A	Multiple sclerosis
	*EDF1*	EC	Endothelial differentiation related factor 1	Neurodegenerative disease
	*NARS*	EC	Asparaginyl-tRNA synthetase	Neurodegenerative disease
Up-regulated	*SERPINA3*	EC	Serpin family A member 3	AD
	*SLC16A9*	EC	Solute carrier family 16 member 9	AD
	*TAC1*	EC	Tachykinin precursor 1	AD
	*GFAP*	EC	Glial fibrillary acidic protein	AD
	*MAN2A1*	EC	Mannosidase alpha class 2A member 1	AD
	*SRSF6*	EC	Serine and arginine rich splicing factor 6	AD
	*HSPB8*	EC	Heat shock protein family B (small) member 8	AD
	*MEGF10*	EC	Multiple EGF like domains 10	AD
	*PRDX1*	EC	Peroxiredoxin 1	AD
	*CP*	EC	Ceruloplasmin	AD
	*PRDX6*	EC	Peroxiredoxin 6	AD
	*CAPN2*	EC	Calpain 2	AD
	*PLXDC2*	EC	Plexin domain containing 2	AD
	*IGSF6*	EC	Immunoglobulin superfamily member 6	AD
	*RAB10*	EC	RAB10, member RAS oncogene family	AD
	*ADAP2*	EC	ArfGAP with dual PH domains 2	AD
	*NPL*	EC	N-acetylneuraminate pyruvate lyase	AD
	*LAP3*	EC	Leucine aminopeptidase 3	AD
	*ATRAID*	EC	All-trans retinoic acid induced differentiation factor	AD
	*APBB1IP*	EC	Amyloid beta precursor protein binding family B member 1 interacting protein	AD
	*DYNLT1*	EC	Dynein light chain Tctex-type 1	AD
	*ARFGAP3*	EC	ADP ribosylation factor GTPase activating protein 3	AD
	*FAM133B*	EC	Family with sequence similarity 133 member B	AD
	*IQCK*	EC	IQ motif containing K	AD
	*WDR11*	EC	WD repeat domain 11	AD
	*CLU*	EC	Clusterin	AD
	*B2M*	EC	Beta-2-microglobulin	AD
	*MT3*	MTG	Metallothionein 3	AD
	*SEPP1*	MTG	Selenoprotein P	AD
	*NME2*	MTG	NME/NM23 nucleoside diphosphate kinase 2	AD
	*EEF2*	MTG	Eukaryotic translation elongation factor 2	AD
	*PCSK1N*	HIP	Proprotein convertase subtilisin/kexin type 1 inhibitor	AD
	*MT1M*	HIP	Metallothionein 1M	AD
	*GJA1*	HIP	Gap junction protein alpha 1	AD
	*STARD7*	EC	StAR related lipid transfer domain containing 7	PD
	*MYBPC1*	EC	Myosin binding protein C, slow type	PD
	*AMOTL2*	EC	Angiomotin like 2	PD
	*VPS13C*	EC	Vacuolar protein sorting 13 homolog C	PD
	*RNF19A*	EC	Ring finger protein 19A, RBR E3 ubiquitin protein ligase	PD
	*NUP133*	EC	Nucleoporin 133	PD
	*PCMTD2*	EC	Protein-L-isoaspartate (D-aspartate) O-methyltransferase domain containing 2	PD
	*HLA-A*	EC	Major histocompatibility complex, class I, A	Multiple sclerosis
	*ZNF536*	EC	Zinc finger protein 536	Multiple sclerosis
	*STOM*	EC	Stomatin	Multiple sclerosis
	*QKI*	EC	QKI, KH domain containing RNA binding	Multiple sclerosis
	*CAPS*	EC	Calcyphosine	Multiple sclerosis
	*SPP1*	EC	Secreted phosphoprotein 1	Multiple sclerosis
	*ACADM*	EC	Acyl-CoA dehydrogenase, C-4 to C-12 straight chain	Multiple sclerosis
	*HLA-DMA*	EC	Major histocompatibility complex, class II, DM alpha	Multiple sclerosis
	*NFASC*	EC	Neurofascin	Multiple sclerosis
	*PLEK*	EC	Pleckstrin	Multiple sclerosis; HD
	*ACTL6A*	EC	Actin like 6A	Multiple sclerosis; PD
	*GTF2H5*	EC	General transcription factor IIH subunit 5	Multiple sclerosis
	*IRF8*	EC	Interferon regulatory factor 8	Multiple sclerosis
	*TRIM22*	EC	Tripartite motif containing 22	Multiple sclerosis
	*PRPF38B*	EC	Pre-mRNA processing factor 38B	Multiple sclerosis
	*KMT5B*	EC	Lysine methyltransferase 5B	Multiple sclerosis
	*AK3*	EC	Adenylate kinase 3	HD
	*PPM1K*	EC	Protein phosphatase, Mg2+/Mn2+ dependent 1K	HD
	*FUT9*	EC	Fucosyltransferase 9	HD
	*FAM189A2*	EC	Family with sequence similarity 189 member A2	Neurodegenerative disease
	*ATG4C*	EC	Autophagy related 4C cysteine peptidase	Neurodegenerative disease
	*TSPAN6*	EC	Tetraspanin 6	Neurodegenerative disease
	*SLC44A1*	EC	Solute carrier family 44 member 1	Neurodegenerative disease
	*SUMF1*	EC	Sulfatase modifying factor 1	Neurodegenerative disease

Table 5The down/up situation of 57 AD-associate genes identified in the meta-analysis in each individual dataset.

**Table:** 

**Down- regulated**	**Hippocampus (HIP)**									
	**GSE5281**		**GSE36980**		**GSE48350**		**GSE29378**		**GSE1297**	
	**log2FoldChange**	***P*-Value**	**log2FoldChange**	***P*-Value**	**log2FoldChange**	***P*-Value**	**log2FoldChange**	***P*-Value**	**log2FoldChange**	***P*-Value**
*RPS27A*	−0.726653445	0.037078998	0.006418053	0.634791602	0.162273062	0.00159256	4.70E-05	0.006604828	0.224013488	0.913570635
**Up-regulated**	**log2FoldChange**	***P*****-Value**	**log2FoldChange**	***P*****-Value**	**log2FoldChange**	***P*****-Value**	**log2FoldChange**	***P*****-Value**	**log2FoldChange**	***P*****-Value**
*PCSK1N*	0.138069789	0.50703397	−0.031361177	0.057833175	−0.233532889	0.187009896	NA		−0.562365704	0.177607105
*MT1M*	0.705082952	0.092700761	0.047028345	0.282787829	0.125837365	0.739204026	0.134012269	0.872273811	−0.57594349	0.430641885
*GJA1*	0.321846385	0.423970204	0.04636156	0.176170552	−0.046712345	0.743685092	-0.017238343	0.444546304	0.03288786	0.614266746

**Table:** 

**Down- regulated**	**Entorhinal Cortex (EC)**					
	**GSE5281**		**GSE26927**		**GSE48350**	
	**log2FoldChange**	***P*****-Value**	**log2FoldChange**	***P*****-Value**	**log2FoldChange**	***P*****-Value**
*ADAP1*	−0.975358771	0.000738556	−0.10598992	0.4322535	0.26177215	0.008467065
*BRSK2*	−2.877946644	2.47*E*−05	−0.05492211	0.6649916	0.471708705	0.012467794
*DMTN*	−1.424808111	8.99*E*−06	0.01319122	0.9421318	0.235990221	0.191857188
*DNM1*	−2.739879787	2.39*E*−07	0.04539906	0.8220179	0.150042132	0.156014246
*FXYD7*	−1.927020252	0.00012567	0.12906713	0.4024216	0.421928539	0.067078593
*GAPDH*	−1.876446535	1.83*E*−05	0.10665021	0.4125155	0.131208665	0.071996601
*GNAS*	−2.168431795	0.003326198	−0.55014104	0.3213458	0.101005549	0.082500384
*GNB5*	−2.629499459	0.000143728	0.05259637	0.6220857	0.267868945	0.177291265
*HCFC1R1*	−2.418575208	0.001577245	0.07272677	0.7672356	0.291574573	0.001166783
*INA*	−2.213790118	0.000286098	0.15823794	0.4368417	0.23025025	0.29758798
*L1CAM*	−2.253719243	3.32*E*−08	0.09255605	0.6813247	0.215178049	0.254031866
*MLST8*	−1.080812867	0.000603178	−0.12108112	0.3325045	0.287724012	0.005909275
*NDUFV3*	−2.456436763	0.000139382	−0.04468396	0.8369119	0.290209615	0.000799514
*NRGN*	−1.93628633	0.004325631	−0.11981346	0.5348417	0.335587722	0.181571319
*PCSK1N*	−2.75543613	6.15*E*−05	0.03089422	0.8535132	0.306834536	0.041926028
*RAD23A*	−1.486686178	1.19*E*−05	−0.06985249	0.4657511	0.155919127	0.023202481
*RPH3A*	−2.205389128	0.000121979	−0.13636354	0.5549691	0.421580071	0.13750631
*SLC17A6*	−1.830552051	0.001580552	−0.03324685	0.816987	0.464266073	0.117376592
*SYN1*	−2.299673462	1.58*E*−05	0.02889328	0.9464393	0.452666293	0.078849751
*TOMM40*	−0.736830295	0.015992849	0.30903301	0.4934736	0.146321059	0.022047574
*TUBB4B*	−2.679987878	0.000319179	0.07067393	0.4499649	0.276553902	0.062125723
**Up-regulated**	**log2FoldChange**	***P*****-Value**	**log2FoldChange**	***P*****-Value**	**log2FoldChange**	***P*****-Value**
*ADAP2*	0.51283287	0.118857833	-0.06659797	0.8099246	0.226833787	0.23714665
*APBB1IP*	0.587584434	0.084938225	−0.00896342	0.9812134	−0.079441481	0.661738945
*ARFGAP3*	1.236283159	0.023286482	0.09021196	0.4114407	−0.035664345	0.74369762
*ATRAID*	0.625394024	0.316212	0.23423602	0.1543085	−0.044280464	0.76558655
*B2M*	1.447935973	0.076662517	−0.34208579	0.2037221	−0.041599563	0.71977472
*CAPN2*	0.84824728	0.053265641	0.17151879	0.2941073	−0.213466413	0.179983124
*CLU*	0.478529519	0.322071446	−0.2573437	0.0879138	−0.024910105	0.955459054
*CP*	0.709886275	0.181039467	−0.78974274	0.1119182	0.101947077	0.871136325
*DYNLT1*	0.818264314	0.079915503	0.05107443	0.725406	−0.020183755	0.897716773
*FAM133B*	0.778470534	0.051976016	−0.21216533	0.0908713	−0.247661748	0.019678966
*GFAP*	0.800783703	0.036506933	−0.21606756	0.5421759	−0.00386905	0.989972713
*HSPB8*	0.496766253	0.077107061	0.04498535	0.8190471	−0.077219912	0.830926271
*IGSF6*	0.501189469	0.262839015	−0.27632074	0.4853182	−0.067940231	0.758869601
*IQCK*	0.333964298	0.13445688	−0.11592661	0.371691	−0.061655779	0.581519573
*LAP3*	0.897642777	0.145483073	−0.24390316	0.2506934	0.010833758	0.940660335
*MAN2A1*	0.747002323	0.172935628	−0.14157596	0.5729284	−0.682271726	0.054038553
*MEGF10*	0.556993686	0.116067563	−0.11060319	0.5623855	−0.336183288	0.089069161
*NPL*	0.446722265	0.131361543	0.00311247	0.9926684	−0.078551165	0.757780969
*PLXDC2*	0.508542748	0.14792643	−0.2940696	0.2734916	−0.234337594	0.176238219
*PRDX1*	0.766030612	0.301038221	−0.10469149	0.3129033	0.000517866	0.995942177
*PRDX6*	0.760314179	0.091473535	−0.08819497	0.5972124	−0.028179809	0.882624237
*RAB10*	0.442205285	0.105401194	−0.00327892	0.9775461	−0.021016368	0.747519625
*SERPINA3*	1.778045885	0.153542154	−0.7074398	0.3106779	0.53138378	0.379307158
*SLC16A9*	−0.083397868	0.891622356	−0.01895744	0.9275386	−0.133889726	0.776238048
*SRSF6*	0.589149827	0.172326071	0.00669309	0.943216	0.126191487	0.600178046
*TAC1*	1.995303687	0.202041997	0.19367133	0.5835214	−0.523156572	0.688917746
*WDR11*	0.811309106	0.060864791	0.13336214	0.3471851	−0.35430392	0.019054577

**Table:** 

**Down- regulated**	**Medial temporal gyrus(MTG)**			
	**GSE5281**		**GSE84422**	
	**log2FoldChange**	***P*****-Value**	**log2FoldChange**	***P*****-Value**
*MT3*	0.471971901	0.06961523	0.006264733	0.376461879
*SEPP1*	0.952243496	0.049773528	0.025532798	0.310596068
*NME2*	0.253272544	0.295414163	0.007540923	0.012257472
*EEF2*	0.450031233	0.0824617	0.000633239	0.600645423

### Analysis of the DEG PPI Network and Identification of Hub Nodes

**Figure 5:**
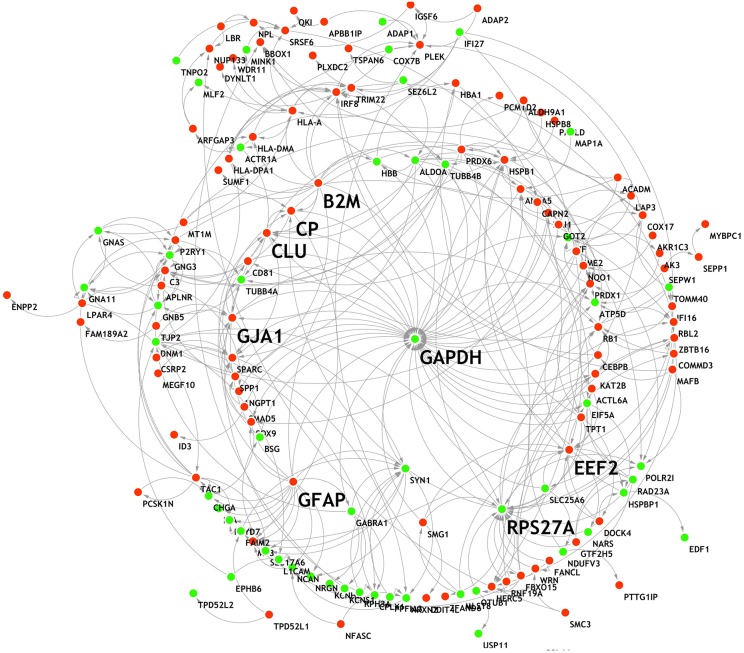
PPI network of the DEGs. Red, up-regulation; green, down-regulation.

The 207 DEGs were subjected to STRING v.11.0 to study the PPI network, and 154 nodes were sorted. The interaction network was then analyzed using the Network Analyzer tool (Shannon et al., 2003) (Figure 5). The 154 genes showed varying degrees of distribution, with a maximum degree of 39 and a minimum degree of 1. The upper eight nodes (top 5% of all nodes) with high degree and high closeness centrality values were chosen as hub nodes (Table 6). These eight hubs (*GAPDH, RPS27A, GFAP, B2M, CLU, EEF2, GJA1*, and *CP*) have all previously been found to be involved in the process of AD (Deane et al., 2005; Li et al., 2005; Olah et al., 2011; El Kadmiri et al., 2014; Guerreiro et al., 2015; Kamphuis et al., 2015; Almeida et al., 2018; Karagkouni et al., 2018).

**Table 6:** The hub genes identified from the meta-analysis DEGs.

**Gene symbols**	**Degree**	**ClosenessCentrality**	**Meta.expression**	**Meta. Qpval**	**Meta. pval**	**Brain tissue**
*GAPDH*	39	0.47727273	−0.543132906	0.903040583	0.011108607	EC
*RPS27A*	25	0.42241379	−0.506389968	0.501831808	0.00902696	HIP
*B2M*	13	0.3878628	0.353023046	0.579830231	0.040853160	EC
*GFAP*	16	0.38582677	0.398018735	0.353484653	0.013506136	EC
*EEF2*	14	0.3828125	0.386796708	0.358680034	0.043927779	MTG
*CP*	10	0.38582677	0.523351329	0.918943259	0.0137236	EC
*CLU*	12	0.38481675	0.447778971	0.448785848	0.01315427	EC
*GJA1*	10	0.3828125	0.252228568	0.674305099	0.045547895	HIP

### The miRNAs Associated With the AD-Associated and Other Neurodegenerative Disease-Associated DEGs, and the lncRNAs Associated With These miRNAs

To investigate the interactions between the AD-associated DEGs and non-coding RNAs, miRNAs associated with these genes were analyzed using the DIANA-Tarbase v.8.0 database. Of these miRNAs, 48 miRNAs were related to AD (Table 7), and 22 miRNAs were associated with other neurodegenerative diseases, such as multiple sclerosis and Parkinson's disease (Table 8). To investigate the interactions between the other neurodegenerative disease-associated DEGs and non-coding RNAs, 17 miRNAs were identified as being related to AD (Table 9). Moreover, most of these miRNAs were in turn regulated by many lncRNAs.

**Table 7:** AD-associate miRNAs associated with the AD-associate DEGs.

**AD-associate DEGs**	**AD-associate miRNAs associated with genes**	**lncRNAs associated with miRNAs**
*ADAP1* (downregulated)	hsa-miR-21-3p	XLOC_013174
	hsa-miR-27a-5p	
*BRSK2* (downregulated)	hsa-miR-27a-3p	C1orf132;DLX6-AS1;FLJ37201;IPW;KCNA3;KCNQ1OT1;LINC00548;LINC00662;LOC283070;MIR4534;NEAT1;RASSF8-AS1;RP11-111K18.2;RP11-129M16.4;RP11-175O19.4;RP11-196G18.22;RP11-314B1.2;RP11-361F15.2;RP11-553L6.5;RP11-94L15.2;RP13-735L24.1;RPA3-AS1;SNHG14;TMX2-CTNND1;TOB1-AS1;XLOC_003240;XLOC_008152;XLOC_010463;XLOC_011185;XLOC_013093;XXbac-BPGBPG55C20.2
*DMTN* (downregulated)	hsa-miR-124-3p	AL022344.7;ERVK13-1;KCNQ1OT1;LINC00643;LOC284581;NEAT1;RAD51L3-RFFL;RP11-508N22.12;RP11-731J8.2;RP11-74E22.8;TMEM256-PLSCR3;TTTY15;XLOC_006753;XLOC_010853;XLOC_013174;XLOC_013844
	hsa-miR-27a-3p	C1orf132;DLX6-AS1;FLJ37201;IPW;KCNA3;KCNQ1OT1;LINC00548;LINC00662;LOC283070;MIR4534;NEAT1;RASSF8-AS1;RP11-111K18.2;RP11-129M16.4;RP11-175O19.4;RP11-196G18.22;RP11-314B1.2;RP11-361F15.2;RP11-553L6.5;RP11-94L15.2;RP13-735L24.1;RPA3-AS1;SNHG14;TMX2-CTNND1;TOB1-AS1;XLOC_003240;XLOC_008152;XLOC_010463;XLOC_011185;XLOC_013093;XXbac-BPGBPG55C20.2
*DNM1* (downregulated)	hsa-miR-124-3p	AL022344.7;ERVK13-1;KCNQ1OT1;LINC00643;LOC284581;NEAT1;RAD51L3-RFFL;RP11-508N22.12;RP11-731J8.2;RP11-74E22.8;TMEM256-PLSCR3;TTTY15;XLOC_006753;XLOC_010853;XLOC_013174;XLOC_013844
	hsa-miR-128-3p	
	hsa-miR-27a-3p	C1orf132;DLX6-AS1;FLJ37201;IPW;KCNA3;KCNQ1OT1;LINC00548;LINC00662;LOC283070;MIR4534;NEAT1;RASSF8-AS1;RP11-111K18.2;RP11-129M16.4;RP11-175O19.4;RP11-196G18.22;RP11-314B1.2;RP11-361F15.2;RP11-553L6.5;RP11-94L15.2;RP13-735L24.1;RPA3-AS1;SNHG14;TMX2-CTNND1;TOB1-AS1;XLOC_003240;XLOC_008152;XLOC_010463;XLOC_011185;XLOC_013093;XXbac-BPGBPG55C20.2
	hsa-miR-9-5p	CTB-89H12.4;KCNQ1OT1;RP11-273G15.2;RP11-793H13.8;SNHG14;TSNAX-DISC1;TUG1;XLOC_013093
*GNAS* (downregulated)	hsa-miR-182-5p	HOXA10-HOXA9;KCNQ1OT1;PCAT19;RP1-309I22.2
	hsa-miR-424-5p	AC005540.3;C1orf132;C1RL-AS1;INO80B-WBP1;KCNQ1OT1;LINC00662;MIA-RAB4B;RP11-379I19.1;RP1-309I22.2;RP5-991G20.1;RP6-24A23.7;XIST;XLOC_006753;XLOC_008207
*GNB5* (downregulated)	hsa-miR-124-3p	AL022344.7;ERVK13-1;KCNQ1OT1;LINC00643;LOC284581;NEAT1;RAD51L3-RFFL;RP11-508N22.12;RP11-731J8.2;RP11-74E22.8;TMEM256-PLSCR3;TTTY15;XLOC_006753;XLOC_010853;XLOC_013174;XLOC_013844
	hsa-miR-148b-3p	CASC7;OIP5-AS1;SLMO2-ATP5E;SNHG14
	hsa-miR-503-5p	
*HCFC1R1* (downregulated)	hsa-miR-124-3p	AL022344.7;ERVK13-1;KCNQ1OT1;LINC00643;LOC284581;NEAT1;RAD51L3-RFFL;RP11-508N22.12;RP11-731J8.2;RP11-74E22.8;TMEM256-PLSCR3;TTTY15;XLOC_006753;XLOC_010853;XLOC_013174;XLOC_013844
	hsa-miR-23b-3p	CASC7;CTC-459F4.3;KCNQ1OT1;NEAT1;RP11-159G9.5;RP11-215G15.5;SNHG14;TOB1-AS1;XIST;XLOC_005784;ZNRD1-AS1
	hsa-miR-27a-5p	
*HSPBP1* (downregulated)	hsa-miR-27a-3p	C1orf132;DLX6-AS1;FLJ37201;IPW;KCNA3;KCNQ1OT1;LINC00548;LINC00662;LOC283070;MIR4534;NEAT1;RASSF8-AS1;RP11-111K18.2;RP11-129M16.4;RP11-175O19.4;RP11-196G18.22;RP11-314B1.2;RP11-361F15.2;RP11-553L6.5;RP11-94L15.2;RP13-735L24.1;RPA3-AS1;SNHG14;TMX2-CTNND1;TOB1-AS1;XLOC_003240;XLOC_008152;XLOC_010463;XLOC_011185;XLOC_013093;XXbac-BPGBPG55C20.2
*INA* (downregulated)	hsa-let-7b-5p	AP001055.6;BACE1-AS;CASC7;HOXA10-HOXA9;KCNQ1OT1;NEAT1;RP11-23J9.4;RP11-391M1.4;RP11-438B23.2;RP11-834C11.4;RP11-923I11.8;ST3GAL5-AS1;TRG-AS1;TUG1;XIST;XLOC_000647
	hsa-miR-16-5p	AC005540.3;FGF14-IT1;GS1-358P8.4;LINC00662;RP11-359B12.2;RP11-361F15.2;RP11-96D1.10;RP3-508I15.20;RP6-24A23.7;XLOC_003546;XLOC_006753;XLOC_008207;XLOC_013174
*L1CAM* (downregulated)	hsa-let-7b-5p	AP001055.6;BACE1-AS;CASC7;HOXA10-HOXA9;KCNQ1OT1;NEAT1;RP11-23J9.4;RP11-391M1.4;RP11-438B23.2;RP11-834C11.4;RP11-923I11.8;ST3GAL5-AS1;TRG-AS1;TUG1;XIST;XLOC_000647
	hsa-miR-124-3p	AL022344.7;ERVK13-1;KCNQ1OT1;LINC00643;LOC284581;NEAT1;RAD51L3-RFFL;RP11-508N22.12;RP11-731J8.2;RP11-74E22.8;TMEM256-PLSCR3;TTTY15;XLOC_006753;XLOC_010853;XLOC_013174;XLOC_013844
	hsa-miR-16-5p	AC005540.3;FGF14-IT1;GS1-358P8.4;LINC00662;RP11-359B12.2;RP11-361F15.2;RP11-96D1.10;RP3-508I15.20;RP6-24A23.7;XLOC_003546;XLOC_006753;XLOC_008207;XLOC_013174
	hsa-miR-182-5p	HOXA10-HOXA9;KCNQ1OT1;PCAT19;RP1-309I22.2
	hsa-miR-195-5p	AC005540.3;FGF14-IT1;GS1-358P8.4;KCNQ1OT1;LINC00662;RP11-361F15.2;RP11-96D1.10;RP5-991G20.1;RP6-24A23.7;XLOC_006753;XLOC_008207;XLOC_013174
	hsa-miR-375	KCNQ1OT1;SNHG14;SNORD116-20
*MLST8* (downregulated)	hsa-let-7b-5p	AP001055.6;BACE1-AS;CASC7;HOXA10-HOXA9;KCNQ1OT1;NEAT1;RP11-23J9.4;RP11-391M1.4;RP11-438B23.2;RP11-834C11.4;RP11-923I11.8;ST3GAL5-AS1;TRG-AS1;TUG1;XIST;XLOC_000647
	hsa-miR-124-3p	AL022344.7;ERVK13-1;KCNQ1OT1;LINC00643;LOC284581;NEAT1;RAD51L3-RFFL;RP11-508N22.12;RP11-731J8.2;RP11-74E22.8;TMEM256-PLSCR3;TTTY15;XLOC_006753;XLOC_010853;XLOC_013174;XLOC_013844
	hsa-miR-130a-3p	CASC7;H19;SNHG14
	hsa-miR-200b-3p	CTC-444N24.11;XIST;XLOC_013174
*NDUFV3* (downregulated)	hsa-let-7a-5p	AP001055.6;BACE1-AS;CASC7;HOXA10-HOXA9;KCNQ1OT1;MEG3;NEAT1;RP11-391M1.4;RP11-438B23.2;RP11-834C11.4;RP11-923I11.8;ST3GAL5-AS1;TRG-AS1;TUG1;XIST;XLOC_008829;XLOC_010445;XLOC_013274;ZNRD1-AS1
	hsa-let-7b-5p	AP001055.6;BACE1-AS;CASC7;HOXA10-HOXA9;KCNQ1OT1;NEAT1;RP11-23J9.4;RP11-391M1.4;RP11-438B23.2;RP11-834C11.4;RP11-923I11.8;ST3GAL5-AS1;TRG-AS1;TUG1;XIST;XLOC_000647
	hsa-let-7c-5p	CASC7;TRG-AS1;XIST;XLOC_010445
*NRGN* (downregulated)	hsa-miR-107	CASC7;KCNQ1OT1;LINC00662;MIR4534;RP11-361F15.2;RP6-24A23.7;STAG3L5P-PVRIG2P-PILRB;XLOC_006753
	hsa-miR-195-5p	AC005540.3;FGF14-IT1;GS1-358P8.4;KCNQ1OT1;LINC00662;RP11-361F15.2;RP11-96D1.10;RP5-991G20.1;RP6-24A23.7;XLOC_006753;XLOC_008207;XLOC_013174
	hsa-miR-26a-5p	CTD-3064H18.1;DLX6-AS1;GAS5;GS1-124K5.3;KCNQ1OT1;MIR181A1HG;RP11-1006G14.4;RP11-119F7.5;RP11-120E11.2;RP11-1C8.7;RP11-282O18.3;RP11-305E6.4;RP11-78O7.2;RP4-635E18.8;RP5-1172N10.4;THUMPD3-AS1;TUG1;VSTM2A-OT1;XLOC_001148;XLOC_002746;XLOC_013174
*PCSK1N* (downregulated)	hsa-miR-124-3p	AL022344.7;ERVK13-1;KCNQ1OT1;LINC00643;LOC284581;NEAT1;RAD51L3-RFFL;RP11-508N22.12;RP11-731J8.2;RP11-74E22.8;TMEM256-PLSCR3;TTTY15;XLOC_006753;XLOC_010853;XLOC_013174;XLOC_013844
*RAD23A* (downregulated)	hsa-miR-124-3p	AL022344.7;ERVK13-1;KCNQ1OT1;LINC00643;LOC284581;NEAT1;RAD51L3-RFFL;RP11-508N22.12;RP11-731J8.2;RP11-74E22.8;TMEM256-PLSCR3;TTTY15;XLOC_006753;XLOC_010853;XLOC_013174;XLOC_013844
	hsa-miR-200b-3p	CTC-444N24.11;XIST;XLOC_013174
*RPH3A* (downregulated)	hsa-miR-27a-3p	C1orf132;DLX6-AS1;FLJ37201;IPW;KCNA3;KCNQ1OT1;LINC00548;LINC00662;LOC283070;MIR4534;NEAT1;RASSF8-AS1;RP11-111K18.2;RP11-129M16.4;RP11-175O19.4;RP11-196G18.22;RP11-314B1.2;RP11-361F15.2;RP11-553L6.5;RP11-94L15.2;RP13-735L24.1;RPA3-AS1;SNHG14;TMX2-CTNND1;TOB1-AS1;XLOC_003240;XLOC_008152;XLOC_010463;XLOC_011185;XLOC_013093;XXbac-BPGBPG55C20.2
*RPS27A* (downregulated)	hsa-miR-181a-5p	AC000403.4;CASC7;CTB-89H12.4;IPW;KCNIP4-IT1;KCNQ1OT1;LINC00506;N4BP2L2-IT2;RP11-10E18.7;RP11-1134I14.8;RP11-147L13.14;RP11-314B1.2;RP11-361F15.2;RP11-707A18.1;RP1-309I22.2;XLOC_003971;XLOC_010463;XLOC_011185;ZNF883;ZNRD1-AS1
*SLC17A6* (downregulated)	hsa-miR-27a-3p	C1orf132;DLX6-AS1;FLJ37201;IPW;KCNA3;KCNQ1OT1;LINC00548;LINC00662;LOC283070;MIR4534;NEAT1;RASSF8-AS1;RP11-111K18.2;RP11-129M16.4;RP11-175O19.4;RP11-196G18.22;RP11-314B1.2;RP11-361F15.2;RP11-553L6.5;RP11-94L15.2;RP13-735L24.1;RPA3-AS1;SNHG14;TMX2-CTNND1;TOB1-AS1;XLOC_003240;XLOC_008152;XLOC_010463;XLOC_011185;XLOC_013093;XXbac-BPGBPG55C20.2
*TOMM40* (downregulated)	hsa-miR-124-3p	AL022344.7;ERVK13-1;KCNQ1OT1;LINC00643;LOC284581;NEAT1;RAD51L3-RFFL;RP11-508N22.12;RP11-731J8.2;RP11-74E22.8;TMEM256-PLSCR3;TTTY15;XLOC_006753;XLOC_010853;XLOC_013174;XLOC_013844
*TUBB4B* (downregulated)	hsa-let-7b-5p	AP001055.6;BACE1-AS;CASC7;HOXA10-HOXA9;KCNQ1OT1;NEAT1;RP11-23J9.4;RP11-391M1.4;RP11-438B23.2;RP11-834C11.4;RP11-923I11.8;ST3GAL5-AS1;TRG-AS1;TUG1;XIST;XLOC_000647
	hsa-miR-128-3p	
	hsa-miR-148b-3p	CASC7;CTD-2303H24.2;OIP5-AS1;SLMO2-ATP5E;SNHG14
	hsa-miR-16-5p	AC005540.3;FGF14-IT1;GS1-358P8.4;KCNQ1OT1;LINC00662;RP11-359B12.2;RP11-361F15.2;RP11-96D1.10;RP3-508I15.20;RP6-24A23.7;XLOC_003546;XLOC_006753;XLOC_008207;XLOC_013174
	hsa-miR-17-5p	AC006548.28;CTB-89H12.4;CTD-2015H6.3;GABPB1-AS1;HCG11;LINC00657;MIR6080;MIR8072;PWAR6;PWARSN;RP11-162A12.4;RP11-171I2.1;RP11-361F15.2;RP11-363E7.4;RP11-399O19.9;RP11-553L6.5;RP11-81A1.6;RP11-909M7.3;XLOC_011677
	hsa-miR-18a-5p	AC000403.4;CASC7;CTB-89H12.4;IPW;KCNIP4-IT1;KCNQ1OT1;LINC00506;N4BP2L2-IT2;RP11-10E18.7;RP11-1134I14.8;RP11-147L13.14;RP11-314B1.2;RP11-361F15.2;RP11-707A18.1;RP1-309I22.2;XLOC_003971;XLOC_010463;XLOC_011185;ZNF883;ZNRD1-AS1
	hsa-miR-18b-5p	XIST;XLOC_014102
	hsa-miR-23a-3p	CASC7;KCNQ1OT1;NEAT1;RP11-159G9.5;RP11-215G15.5;SNHG14;TOB1-AS1;XIST;ZNRD1-AS1
	hsa-miR-23b-3p	CASC7;CTC-459F4.3;KCNQ1OT1;NEAT1;RP11-159G9.5;RP11-215G15.5;SNHG14;TOB1-AS1;XIST;XLOC_005784;ZNRD1-AS1
	hsa-miR-27b-3p	C1orf132;DLX6-AS1;FLJ37201;IPW;KCNA3;KCNQ1OT1;LINC00548;LINC00662;LOC283070;NEAT1;RASSF8-AS1;RP11-111K18.2;RP11-129M16.4;RP11-175O19.4;RP11-196G18.22;RP11-314B1.2;RP11-553L6.5;RP11-94L15.2;RP13-735L24.1;RPA3-AS1;SNHG14;
*ADAP2* (upregulated)	hsa-miR-16-5p	AC005540.3;FGF14-IT1;GS1-358P8.4;KCNQ1OT1;LINC00662;RP11-359B12.2;RP11-361F15.2;RP11-96D1.10;RP3-508I15.20;RP6-24A23.7;XLOC_003546;XLOC_006753;XLOC_008207;XLOC_013174
	hsa-miR-29c-3p	AC005154.6;AC006548.28;H19;KCNQ1OT1;LINC00674;MIR4697HG;NEAT1;RP11-314B1.2;RP11-582E3.6;RP4-630A11.3;THUMPD3-AS1;TTTY15;TUG1;XLOC_004366;XLOC_007942;XLOC_008295
	hsa-let-7b-5p	AP001055.6;BACE1-AS;CASC7;HOXA10-HOXA9;KCNQ1OT1;NEAT1;RP11-23J9.4;RP11-391M1.4;RP11-438B23.2;RP11-834C11.4;RP11-923I11.8;ST3GAL5-AS1;TRG-AS1;TUG1;XIST;XLOC_000647
	hsa-miR-101-3p	AC005235.1;CTD-2303H24.2;CTD-2571L23.8;FAM201A;HCG11;KCNQ1OT1;LINC00657;LINC00662;LINC00936;RP11-102L12.2;RP11-1134I14.8;RP11-196G18.24;RP11-350F4.2;RP11-378J18.8;RP11-421E14.2;XIST;XLOC_002872
*APBB1IP* (upregulated)	hsa-miR-27a-3p	C1orf132;DLX6-AS1;FLJ37201;IPW;KCNA3;KCNQ1OT1;LINC00548;LINC00662;LOC283070;MIR4534;NEAT1;RASSF8-AS1;RP11-111K18.2;RP11-129M16.4;RP11-175O19.4;RP11-196G18.22;RP11-314B1.2;RP11-361F15.2;RP11-553L6.5;RP11-94L15.2;RP13-735L24.1;RPA3-AS1;SNHG14;TMX2-CTNND1;TOB1-AS1;XLOC_003240;XLOC_008152;XLOC_010463;XLOC_011185;XLOC_013093;XXbac-BPGBPG55C20.2
*ARFGAP3* (upregulated)	hsa-miR-200b-3p	CTC-444N24.11;XIST;XLOC_013174
*ATRAID* (upregulated)	hsa-miR-23b-3p	CASC7;CTC-459F4.3;KCNQ1OT1;NEAT1;RP11-159G9.5;RP11-215G15.5;SNHG14;TOB1-AS1;XIST;XLOC_005784;ZNRD1-AS1
	hsa-let-7b-5p	AP001055.6;BACE1-AS;CASC7;HOXA10-HOXA9;KCNQ1OT1;NEAT1;RP11-23J9.4;RP11-391M1.4;RP11-438B23.2;RP11-834C11.4;RP11-923I11.8;ST3GAL5-AS1;TRG-AS1;TUG1;XIST;XLOC_000647
	hsa-miR-101-3p	AC005235.1;CTD-2303H24.2;CTD-2571L23.8;FAM201A;HCG11;KCNQ1OT1;LINC00657;LINC00662;LINC00936;RP11-102L12.2;RP11-1134I14.8;RP11-196G18.24;RP11-350F4.2;RP11-378J18.8;RP11-421E14.2;XIST;XLOC_002872
	hsa-miR-16-5p	AC005540.3;FGF14-IT1;GS1-358P8.4;KCNQ1OT1;LINC00662;RP11-359B12.2;RP11-361F15.2;RP11-96D1.10;RP3-508I15.20;RP6-24A23.7;XLOC_003546;XLOC_006753;XLOC_008207;XLOC_013174
*CAPN2* (upregulated)	hsa-miR-101-3p	AC005235.1;CTD-2303H24.2;CTD-2571L23.8;FAM201A;HCG11;KCNQ1OT1;LINC00657;LINC00662;LINC00936;RP11-102L12.2;RP11-1134I14.8;RP11-196G18.24;RP11-350F4.2;RP11-378J18.8;RP11-421E14.2;XIST;XLOC_002872
	hsa-miR-148b-3p	CASC7;CTD-2303H24.2;OIP5-AS1;SLMO2-ATP5E;SNHG14
	hsa-miR-124-3p	AL022344.7;ERVK13-1;KCNQ1OT1;LINC00643;LOC284581;NEAT1;RAD51L3-RFFL;RP11-508N22.12;RP11-731J8.2;RP11-74E22.8;TMEM256-PLSCR3;TTTY15;XLOC_006753;XLOC_010853;XLOC_013174;XLOC_013844
	hsa-miR-101-3p	AC005235.1;CTD-2303H24.2;CTD-2571L23.8;FAM201A;HCG11;KCNQ1OT1;LINC00657;LINC00662;LINC00936;RP11-102L12.2;RP11-1134I14.8;RP11-196G18.24;RP11-350F4.2;RP11-378J18.8;RP11-421E14.2;XIST;XLOC_002872
*CP* (upregulated)	hsa-miR-124-3p	AL022344.7;ERVK13-1;KCNQ1OT1;LINC00643;LOC284581;NEAT1;RAD51L3-RFFL;RP11-508N22.12;RP11-731J8.2;RP11-74E22.8;TMEM256-PLSCR3;TTTY15;XLOC_006753;XLOC_010853;XLOC_013174;XLOC_013844
	hsa-miR-182-5p	HOXA10-HOXA9;KCNQ1OT1;PCAT19;RP1-309I22.2
*DYNLT1* (upregulated)	hsa-miR-23b-3p	CASC7;CTC-459F4.3;KCNQ1OT1;NEAT1;RP11-159G9.5;RP11-215G15.5;SNHG14;TOB1-AS1;XIST;XLOC_005784;ZNRD1-AS1
	hsa-miR-128-3p	
	hsa-miR-124-3p	AL022344.7;ERVK13-1;KCNQ1OT1;LINC00643;LOC284581;NEAT1;RAD51L3-RFFL;RP11-508N22.12;RP11-731J8.2;RP11-74E22.8;TMEM256-PLSCR3;TTTY15;XLOC_006753;XLOC_010853;XLOC_013174;XLOC_013844
*GFAP* (upregulated)	hsa-miR-107	CASC7;KCNQ1OT1;LINC00662;MIR4534;RP11-361F15.2;RP6-24A23.7;STAG3L5P-PVRIG2P-PILRB;XLOC_006753
	hsa-miR-497-5p	AC005540.3;C1orf132;C1RL-AS1;FGF14-IT1;GS1-358P8.4;INO80B-WBP1;KCNQ1OT1;LINC00662;MIA-RAB4B;RP11-361F15.2;RP5-991G20.1;RP6-24A23.7;XIST;XLOC_006753;XLOC_008207;XLOC_013174;XLOC_013424
	hsa-miR-124-3p	AL022344.7;ERVK13-1;KCNQ1OT1;LINC00643;LOC284581;NEAT1;RAD51L3-RFFL;RP11-508N22.12;RP11-731J8.2;RP11-74E22.8;TMEM256-PLSCR3;TTTY15;XLOC_006753;XLOC_010853;XLOC_013174;XLOC_013844
	hsa-miR-15a-5p	AC005540.3;FGF14-IT1;GS1-358P8.4;KCNQ1OT1;LINC00662;MCM3AP-AS1;RP11-361F15.2;RP11-96D1.10;RP3-508I15.20;RP5-991G20.1;RP6-24A23.7;XLOC_003546;XLOC_008207;XLOC_006753;XLOC_013174
	hsa-miR-15b-5p	AC005540.3;FGF14-IT1;GS1-358P8.4;KCNQ1OT1;LINC00662;RP11-361F15.2;RP11-96D1.10;RP3-508I15.20;RP6-24A23.7;XLOC_008207;XLOC_013174
	hsa-miR-16-5p	AC005540.3;FGF14-IT1;GS1-358P8.4;KCNQ1OT1;LINC00662;RP11-359B12.2;RP11-361F15.2;RP11-96D1.10;RP3-508I15.20;RP6-24A23.7;XLOC_003546;XLOC_006753;XLOC_008207;XLOC_013174
	hsa-miR-195-5p	AC005540.3;FGF14-IT1;GS1-358P8.4;KCNQ1OT1;LINC00662;RP11-361F15.2;RP11-96D1.10;RP5-991G20.1;RP6-24A23.7;XLOC_006753;XLOC_008207;XLOC_013174
	hsa-miR-24-3p	CTA-292E10.9;CTC-273B12.8;GABPB1-AS1;LINC00662;LOC388692;MIR4534;RP11-54O7.1;XLOC_006242;XLOC_008461;XLOC_011313
*HSPB8* (upregulated)	hsa-miR-124-3p	AL022344.7;ERVK13-1;KCNQ1OT1;LINC00643;LOC284581;NEAT1;RAD51L3-RFFL;RP11-508N22.12;RP11-731J8.2;RP11-74E22.8;TMEM256-PLSCR3;TTTY15;XLOC_006753;XLOC_010853;XLOC_013174;XLOC_013844
	hsa-let-7b-5p	AP001055.6;BACE1-AS;CASC7;HOXA10-HOXA9;KCNQ1OT1;NEAT1;RP11-23J9.4;RP11-391M1.4;RP11-438B23.2;RP11-834C11.4;RP11-923I11.8;ST3GAL5-AS1;TRG-AS1;TUG1;XIST;XLOC_000647
	hsa-miR-16-5p	AC005540.3;FGF14-IT1;GS1-358P8.4;KCNQ1OT1;LINC00662;RP11-359B12.2;RP11-361F15.2;RP11-96D1.10;RP3-508I15.20;RP6-24A23.7;XLOC_003546;XLOC_006753;XLOC_008207;XLOC_013174
	hsa-miR-133a-3p	
*IQCK* (upregulated)	hsa-miR-124-3p	AL022344.7;ERVK13-1;KCNQ1OT1;LINC00643;LOC284581;NEAT1;RAD51L3-RFFL;RP11-508N22.12;RP11-731J8.2;RP11-74E22.8;TMEM256-PLSCR3;TTTY15;XLOC_006753;XLOC_010853;XLOC_013174;XLOC_013844
	hsa-miR-107	CASC7;KCNQ1OT1;LINC00662;MIR4534;RP11-361F15.2;RP6-24A23.7;STAG3L5P-PVRIG2P-PILRB;XLOC_006753
*LAP3* (upregulated)	hsa-miR-495-3p	
	hsa-miR-503-5p	
	hsa-miR-124-3p	AL022344.7;ERVK13-1;KCNQ1OT1;LINC00643;LOC284581;NEAT1;RAD51L3-RFFL;RP11-508N22.12;RP11-731J8.2;RP11-74E22.8;TMEM256-PLSCR3;TTTY15;XLOC_006753;XLOC_010853;XLOC_013174;XLOC_013844
	hsa-miR-128-3p	
	hsa-miR-16-5p	AC005540.3;FGF14-IT1;GS1-358P8.4;KCNQ1OT1;LINC00662;RP11-359B12.2;RP11-361F15.2;RP11-96D1.10;RP3-508I15.20;RP6-24A23.7;XLOC_003546;XLOC_006753;XLOC_008207;XLOC_013174
	hsa-miR-21-3p	XLOC_013174
	hsa-miR-27a-5p	
	hsa-miR-133a-3p	
*MAN2A1* (upregulated)	hsa-miR-27a-3p	C1orf132;DLX6-AS1;FLJ37201;IPW;KCNA3;KCNQ1OT1;LINC00548;LINC00662;LOC283070;MIR4534;NEAT1;RASSF8-AS1;RP11-111K18.2;RP11-129M16.4;RP11-175O19.4;RP11-196G18.22;RP11-314B1.2;RP11-361F15.2;RP11-553L6.5;RP11-94L15.2;RP13-735L24.1;RPA3-AS1;SNHG14;TMX2-CTNND1;TOB1-AS1;XLOC_003240;XLOC_008152;XLOC_010463;XLOC_011185;XLOC_013093;XXbac-BPGBPG55C20.2
	hsa-miR-27b-3p	C1orf132;DLX6-AS1;FLJ37201;IPW;KCNA3;KCNQ1OT1;LINC00548;LINC00662;LOC283070;NEAT1;RASSF8-AS1;RP11-111K18.2;RP11-129M16.4;RP11-175O19.4;RP11-196G18.22;RP11-314B1.2;RP11-553L6.5;RP11-94L15.2;RP13-735L24.1;RPA3-AS1;SNHG14
	hsa-miR-124-3p	AL022344.7;ERVK13-1;KCNQ1OT1;LINC00643;LOC284581;NEAT1;RAD51L3-RFFL;RP11-508N22.12;RP11-731J8.2;RP11-74E22.8;TMEM256-PLSCR3;TTTY15;XLOC_006753;XLOC_010853;XLOC_013174;XLOC_013844
	hsa-let-7b-5p	AP001055.6;BACE1-AS;CASC7;HOXA10-HOXA9;KCNQ1OT1;NEAT1;RP11-23J9.4;RP11-391M1.4;RP11-438B23.2;RP11-834C11.4;RP11-923I11.8;ST3GAL5-AS1;TRG-AS1;TUG1;XIST;XLOC_000647
	hsa-miR-182-5p	HOXA10-HOXA9;KCNQ1OT1;PCAT19;RP1-309I22.2
*MEGF10* (upregulated)	hsa-miR-26a-5p	CTD-3064H18.1;DLX6-AS1;GAS5;GS1-124K5.3;KCNQ1OT1;MIR181A1HG;RP11-1006G14.4;RP11-119F7.5;RP11-120E11.2;RP11-1C8.7;RP11-282O18.3;RP11-305E6.4;RP11-78O7.2;RP4-635E18.8;RP5-1172N10.4;THUMPD3-AS1;TUG1;VSTM2A-OT1;XLOC_001148;XLOC_002746;XLOC_013174
	hsa-miR-101-3p	AC005235.1;CTD-2303H24.2;CTD-2571L23.8;FAM201A;HCG11;KCNQ1OT1;LINC00657;LINC00662;LINC00936;RP11-102L12.2;RP11-1134I14.8;RP11-196G18.24;RP11-350F4.2;RP11-378J18.8;RP11-421E14.2;XIST;XLOC_002872
	hsa-miR-182-5p	HOXA10-HOXA9;KCNQ1OT1;PCAT19;RP1-309I22.2
*MT1M* (upregulated)	hsa-miR-124-3p	AL022344.7;ERVK13-1;KCNQ1OT1;LINC00643;LOC284581;NEAT1;RAD51L3-RFFL;RP11-508N22.12;RP11-731J8.2;RP11-74E22.8;TMEM256-PLSCR3;TTTY15;XLOC_006753;XLOC_010853;XLOC_013174;XLOC_013844
	hsa-miR-21-3p	XLOC_013174
	hsa-miR-27a-3p	C1orf132;DLX6-AS1;FLJ37201;IPW;KCNA3;KCNQ1OT1;LINC00548;LINC00662;LOC283070;MIR4534;NEAT1;RASSF8-AS1;RP11-111K18.2;RP11-129M16.4;RP11-175O19.4;RP11-196G18.22;RP11-314B1.2;RP11-361F15.2;RP11-553L6.5;RP11-94L15.2;RP13-735L24.1;RPA3-AS1;SNHG14;TMX2-CTNND1;TOB1-AS1;XLOC_003240;XLOC_008152;XLOC_010463;XLOC_011185;XLOC_013093;XXbac-BPGBPG55C20.2
	hsa-miR-27a-5p	
	hsa-miR-376a-5p	KCNQ1OT1;SIK3-IT1
*MT3* (upregulated)	hsa-let-7b-5p	AP001055.6;BACE1-AS;CASC7;HOXA10-HOXA9;KCNQ1OT1;NEAT1;RP11-23J9.4;RP11-391M1.4;RP11-438B23.2;RP11-834C11.4;RP11-923I11.8;ST3GAL5-AS1;TRG-AS1;TUG1;XIST;XLOC_000647
	hsa-miR-182-5p	HOXA10-HOXA9;KCNQ1OT1;PCAT19;RP1-309I22.2
*NPL* (upregulated)	hsa-miR-16-5p	AC005540.3;FGF14-IT1;GS1-358P8.4;KCNQ1OT1;LINC00662;RP11-359B12.2;RP11-361F15.2;RP11-96D1.10;RP3-508I15.20;RP6-24A23.7;XLOC_003546;XLOC_006753;XLOC_008207;XLOC_013174
	hsa-miR-27a-5p	
*PCSK1N* (upregulated)	hsa-miR-124-3p	AL022344.7;ERVK13-1;KCNQ1OT1;LINC00643;LOC284581;NEAT1;RAD51L3-RFFL;RP11-508N22.12;RP11-731J8.2;RP11-74E22.8;TMEM256-PLSCR3;TTTY15;XLOC_006753;XLOC_010853;XLOC_013174;XLOC_013844
*PLXDC2* (upregulated)	hsa-miR-29a-3p	AC005154.6;AC006548.28;H19;KCNQ1OT1;LINC00674;MIR4697HG;NEAT1;RP11-314B1.2;RP11-582E3.6;RP4-630A11.3;THUMPD3-AS1;TTTY15;TUG1;XLOC_004366;XLOC_007942;XLOC_008295
	hsa-miR-29b-3p	AC005154.6;AC006548.28;H19;KCNQ1OT1;LINC00674;MIR4697HG;NEAT1;RP11-314B1.2;RP11-582E3.6;RP4-630A11.3;THUMPD3-AS1;TTTY15;TUG1;XLOC_004366;XLOC_007942;XLOC_008295
	hsa-miR-29c-3p	AC005154.6;AC006548.28;H19;KCNQ1OT1;LINC00674;MIR4697HG;NEAT1;RP11-314B1.2;RP11-582E3.6;RP4-630A11.3;THUMPD3-AS1;TTTY15;TUG1;XLOC_004366;XLOC_007942;XLOC_008295
	hsa-miR-27a-3p	AC005154.6;AC006548.28;H19;KCNQ1OT1;LINC00674;MIR4697HG;NEAT1;RP11-314B1.2;RP11-582E3.6;RP4-630A11.3;THUMPD3-AS1;TTTY15;TUG1;XLOC_004366;XLOC_007942;XLOC_008295
*PRDX1* (upregulated)	hsa-miR-29a-3p	AC005154.6;AC006548.28;H19;KCNQ1OT1;LINC00674;MIR4697HG;NEAT1;RP11-314B1.2;RP11-582E3.6;RP4-630A11.3;THUMPD3-AS1;TTTY15;TUG1;XLOC_004366;XLOC_007942;XLOC_008295
	hsa-miR-23b-3p	CASC7;CTC-459F4.3;KCNQ1OT1;NEAT1;RP11-159G9.5;RP11-215G15.5;SNHG14;TOB1-AS1;XIST;XLOC_005784;ZNRD1-AS1
	hsa-miR-375	KCNQ1OT1;SNHG14;SNORD116-20
*PRDX6* (upregulated)	hsa-miR-23b-3p	CASC7;CTC-459F4.3;KCNQ1OT1;NEAT1;RP11-159G9.5;RP11-215G15.5;SNHG14;TOB1-AS1;XIST;XLOC_005784;ZNRD1-AS1
	hsa-miR-124-3p	AL022344.7;ERVK13-1;KCNQ1OT1;LINC00643;LOC284581;NEAT1;RAD51L3-RFFL;RP11-508N22.12;RP11-731J8.2;RP11-74E22.8;TMEM256-PLSCR3;TTTY15;XLOC_006753;XLOC_010853;XLOC_013174;XLOC_013844
	hsa-miR-133a-3p	
*RAB10* (upregulated)	hsa-miR-130b-3p	CASC7;H19;SNHG14
	hsa-miR-148b-3p	CASC7;CTD-2303H24.2;OIP5-AS1;SLMO2-ATP5E;SNHG14
	hsa-miR-107	CASC7;KCNQ1OT1;LINC00662;MIR4534;RP11-361F15.2;RP6-24A23.7;STAG3L5P-PVRIG2P-PILRB;XLOC_006753
	hsa-miR-16-5p;	AC005540.3;FGF14-IT1;GS1-358P8.4;KCNQ1OT1;LINC00662;RP11-359B12.2;RP11-361F15.2;RP11-96D1.10;RP3-508I15.20;RP6-24A23.7;XLOC_003546;XLOC_006753;XLOC_008207;XLOC_013174
	hsa-miR-124-3p	AL022344.7;ERVK13-1;KCNQ1OT1;LINC00643;LOC284581;NEAT1;RAD51L3-RFFL;RP11-508N22.12;RP11-731J8.2;RP11-74E22.8;TMEM256-PLSCR3;TTTY15;XLOC_006753;XLOC_010853;XLOC_013174;XLOC_013844
	hsa-miR-15a-5p	AC005540.3;FGF14-IT1;GS1-358P8.4;KCNQ1OT1;LINC00662;MCM3AP-AS1;RP11-361F15.2;RP11-96D1.10;RP3-508I15.20;RP5-991G20.1;RP6-24A23.7;XLOC_003546;XLOC_008207;XLOC_006753;XLOC_013174
	hsa-miR-15b-5p	AC005540.3;FGF14-IT1;GS1-358P8.4;KCNQ1OT1;LINC00662;RP11-361F15.2;RP11-96D1.10;RP3-508I15.20;RP6-24A23.7;XLOC_008207;XLOC_013174
	hsa-miR-143-3p	AC090587.2;CTB-193M12.3;EEF1E1-BLOC1S5;FLJ31306;GABPB1-AS1;KCNQ1OT1;LINC00662;MESTIT1;OIP5-AS1;PDCD4-AS1;RP11-424G14.1;RP5-1014D13.2
	hsa-miR-30b-5p	AC096772.6;CASC7;CTA-292E10.9;CTB-89H12.4;HCG18;LINC00461;LOC100128288;MIA-RAB4B;OIP5-AS1;PWRN3;RP11-175O19.4;RP11-265E18.1;RP11-361F15.2;RP11-378J18.8;RP11-618G20.1;RP11-731J8.2;RP1-309I22.2;RP6-24A23.7;TRHDE-AS1;UG0898H09;XIST;XLOC_005753;XLOC_008207
	hsa-miR-30c-5p	AC096772.6;CASC7;CTA-292E10.9;CTB-89H12.4;LINC00461;LOC100128288;MIA-RAB4B;PWRN3;RP11-175O19.4;RP11-265E18.1;RP11-361F15.2;RP11-618G20.1;RP11-731J8.2;RP1-309I22.2;UG0898H09;XIST;XLOC_005753;XLOC_008207
	hsa-miR-195-5p	AC005540.3;FGF14-IT1;GS1-358P8.4;KCNQ1OT1;LINC00662;RP11-361F15.2;RP11-96D1.10;RP5-991G20.1;RP6-24A23.7;XLOC_006753;XLOC_008207;XLOC_013174
	hsa-miR-23a-3p	CASC7;KCNQ1OT1;NEAT1;RP11-159G9.5;RP11-215G15.5;SNHG14;TOB1-AS1;XIST;ZNRD1-AS1
	hsa-miR-23b-3p	CASC7;CTC-459F4.3;KCNQ1OT1;NEAT1;RP11-159G9.5;RP11-215G15.5;SNHG14;TOB1-AS1;XIST;XLOC_005784;ZNRD1-AS1
	hsa-miR-497-5p	AC005540.3;C1orf132;C1RL-AS1;FGF14-IT1;GS1-358P8.4;INO80B-WBP1;KCNQ1OT1;LINC00662;MIA-RAB4B;RP11-361F15.2;RP5-991G20.1;RP6-24A23.7;XIST;XLOC_006753;XLOC_008207;XLOC_013174;XLOC_013424
*SEPP1* (upregulated)	hsa-miR-20a-5p	AC006548.28;CTB-89H12.4;LINC00116;GABPB1-AS1;RP11-553L6.5;RP11-81A1.6;SNORD109A;XIST;XLOC_002263;XLOC_004804;XLOC_013093
	hsa-miR-124-3p	AL022344.7;ERVK13-1;KCNQ1OT1;LINC00643;LOC284581;NEAT1;RAD51L3-RFFL;RP11-508N22.12;RP11-731J8.2;RP11-74E22.8;TMEM256-PLSCR3;TTTY15;XLOC_006753;XLOC_010853;XLOC_013174;XLOC_013844
	hsa-let-7b-5p	AP001055.6;BACE1-AS;CASC7;HOXA10-HOXA9;KCNQ1OT1;NEAT1;RP11-23J9.4;RP11-391M1.4;RP11-438B23.2;RP11-834C11.4;RP11-923I11.8;ST3GAL5-AS1;TRG-AS1;TUG1;XIST;XLOC_000647
	hsa-miR-101-3p	AC005235.1;CTD-2303H24.2;CTD-2571L23.8;FAM201A;HCG11;KCNQ1OT1;LINC00657;LINC00662;LINC00936;RP11-102L12.2;RP11-1134I14.8;RP11-196G18.24;RP11-350F4.2;RP11-378J18.8;RP11-421E14.2;XIST;XLOC_002872
	hsa-miR-128-3p	
	hsa-miR-26a-5p	CTD-3064H18.1;DLX6-AS1;GAS5;GS1-124K5.3;KCNQ1OT1;MIR181A1HG;RP11-1006G14.4;RP11-119F7.5;RP11-120E11.2;RP11-1C8.7;RP11-282O18.3;RP11-305E6.4;RP11-78O7.2;RP4-635E18.8;RP5-1172N10.4;THUMPD3-AS1;TUG1;VSTM2A-OT1;XLOC_001148;XLOC_002746;XLOC_013174
*SERPINA3* (upregulated)	hsa-miR-124-3p	AL022344.7;ERVK13-1;KCNQ1OT1;LINC00643;LOC284581;NEAT1;RAD51L3-RFFL;RP11-508N22.12;RP11-731J8.2;RP11-74E22.8;TMEM256-PLSCR3;TTTY15;XLOC_006753;XLOC_010853;XLOC_013174;XLOC_013844
	hsa-miR-20a-5p	AC006548.28;CTB-89H12.4;LINC00116;GABPB1-AS1;RP11-553L6.5;RP11-81A1.6;SNORD109A;XIST;XLOC_002263;XLOC_004804;XLOC_013093
	hsa-miR-30d-5p	CASC7;CTA-292E10.9;CTB-89H12.4;HCG18;LINC00461;LOC100128288;RP11-175O19.4;RP11-361F15.2;RP11-618G20.1;RP1-309I22.2;RP6-24A23.7;XIST;XLOC_008207
	hsa-miR-130a-3p	CASC7;H19;SNHG14
	hsa-miR-182-5p	HOXA10-HOXA9;KCNQ1OT1;PCAT19;RP1-309I22.2
	hsa-miR-27a-3p	AC005154.6;AC006548.28;H19;KCNQ1OT1;LINC00674;MIR4697HG;NEAT1;RP11-314B1.2;RP11-582E3.6;RP4-630A11.3;THUMPD3-AS1;TTTY15;TUG1;XLOC_004366;XLOC_007942;XLOC_008295
*SLC16A9* (upregulated)	hsa-miR-424-5p	AC005540.3;C1orf132;C1RL-AS1;INO80B-WBP1;KCNQ1OT1;LINC00662;MIA-RAB4B;RP11-379I19.1;RP1-309I22.2;RP5-991G20.1;RP6-24A23.7;XIST;XLOC_006753;XLOC_008207;
	hsa-miR-9-3p	ALMS1-IT1;ALMS1-IT1;RP11-175O19.4;RP11-438B23.2;RP4-714D9.5;TTN-AS1;XIST;XLOC_002872;XLOC_010463
	hsa-miR-101-3p	AC005235.1;CTD-2303H24.2;CTD-2571L23.8;FAM201A;HCG11;KCNQ1OT1;LINC00657;LINC00662;LINC00936;RP11-102L12.2;RP11-1134I14.8;RP11-196G18.24;RP11-350F4.2;RP11-378J18.8;RP11-421E14.2;XIST;XLOC_002872
	hsa-miR-21-3p	XLOC_013174
	hsa-miR-27a-5p	
	hsa-miR-29c-3p	AC005154.6;AC006548.28;H19;KCNQ1OT1;LINC00674;MIR4697HG;NEAT1;RP11-314B1.2;RP11-582E3.6;RP4-630A11.3;THUMPD3-AS1;TTTY15;TUG1;XLOC_004366;XLOC_007942;XLOC_008295
*SRSF6* (upregulated)	hsa-miR-26a-5p	CTD-3064H18.1;DLX6-AS1;GAS5;GS1-124K5.3;HOXA10-HOXA9;MIR181A1HG;MIR6080;RP11-1006G14.4;RP11-119F7.5;RP11-120E11.2;RP11-1C8.7;RP11-282O18.3;RP11-305E6.4;RP11-78O7.2;RP4-635E18.8;RP5-1172N10.4;THUMPD3-AS1;VSTM2A-OT1;XLOC_001148;XLOC_002746;XLOC_013174
	hsa-miR-93-5p	AC006548.28;CTB-89H12.4;CTD-2015H6.3;GABPB1-AS1;HCG11;LINC00657;MIR6080;MIR8072;PWAR6;PWARSN;RP11-162A12.4;RP11-361F15.2;RP11-363E7.4;RP11-399O19.9;RP11-553L6.5;RP11-909M7.3;XLOC_004804;XLOC_010706;XLOC_011677
	hsa-miR-340-5p	AC002429.5;CASC7;CTC-444N24.11;LINC00662;LINC01355;NEAT1;RP11-119F7.5;RP11-174G6.5;RP11-96D1.10;TUG1;XIST;XLOC_002282;XLOC_008207
	hsa-miR-137	CASC7;CTB-89H12.4;CTC-459F4.3;HCG18;KB-1410C5.5;OIP5-AS1;RP11-314B1.2;RP11-498C9.15;RP11-78O7.2;SNHG14;XLOC_004457
	hsa-miR-101-3p	AC005235.1;CTD-2303H24.2;CTD-2571L23.8;FAM201A;HCG11;KCNQ1OT1;LINC00657;LINC00662;LINC00936;RP11-102L12.2;RP11-1134I14.8;RP11-196G18.24;RP11-350F4.2;RP11-378J18.8;RP11-421E14.2;XIST;XLOC_002872
	hsa-miR-27a-3p	AC005154.6;AC006548.28;H19;KCNQ1OT1;LINC00674;MIR4697HG;NEAT1;RP11-314B1.2;RP11-582E3.6;RP4-630A11.3;THUMPD3-AS1;TTTY15;TUG1;XLOC_004366;XLOC_007942;XLOC_008295
	hsa-miR-27b-3p	C1orf132;DLX6-AS1;FLJ37201;IPW;KCNA3;KCNQ1OT1;LINC00548;LINC00662;LOC283070;NEAT1;RASSF8-AS1;RP11-111K18.2;RP11-129M16.4;RP11-175O19.4;RP11-196G18.22;RP11-314B1.2;RP11-553L6.5;RP11-94L15.2;RP13-735L24.1;RPA3-AS1;SNHG14;
	hsa-miR-124-3p	AL022344.7;ERVK13-1;KCNQ1OT1;LINC00643;LOC284581;NEAT1;RAD51L3-RFFL;RP11-508N22.12;RP11-731J8.2;RP11-74E22.8;TMEM256-PLSCR3;TTTY15;XLOC_006753;XLOC_010853;XLOC_013174;XLOC_013844
*WDR11* (upregulated)	hsa-miR-26b-5p	GAS5;GS1-124K5.3;HOXA10-HOXA9;RP11-119F7.5;RP11-120E11.2;THUMPD3-AS1;TUG1;XLOC_013174
	hsa-miR-16-5p	AC005540.3;FGF14-IT1;GS1-358P8.4;KCNQ1OT1;LINC00662;RP11-359B12.2;RP11-361F15.2;RP3-508I15.20;RP6-24A23.7;XLOC_003546;XLOC_006753;XLOC_008207;XLOC_013174
	hsa-miR-182-5p	HOXA10-HOXA9;KCNQ1OT1;PCAT19;RP1-309I22.2
*GJA1* (upregulated)	hsa-miR-107	CASC7;KCNQ1OT1;LINC00662;MIR4534;RP11-361F15.2;RP6-24A23.7;STAG3L5P-PVRIG2P-PILRB;XLOC_006753

**Table 8:** Other neurodegenerative disease-specific miRNAs associated with the AD-associate DEGs.

**AD-associate DEGs**	**Other neurodegenerative disease-specific miRNAs associated with genes**	**lncRNAs associated with miRNAs**
*DMTN* (downregulated)	hsa-miR-34a-5p	AC004951.6;AC092535.3;KCNQ1OT1;LINC00662;MIR4534;PCBP2-OT1;RP11-693J15.5
	hsa-miR-7-5p	AC005154.6;DLX6-AS1;KCNQ1OT1;LINC01233;LINC01314;MIR4534;OIP5-AS1;OIP5-AS1;RP11-679B19.1;RP1-309I22.2;XIST
*FXYD7* (downregulated)	hsa-miR-212-3p	CASC7;CTB-89H12.4;NEAT1;RP11-26J3.3;XIST;XLOC_006753
*GAPDH* (downregulated)	hsa-miR-34a-5p	AC004951.6;AC092535.3;KCNQ1OT1;LINC00662;MIR4534;PCBP2-OT1;RP11-693J15.5
*HSPBP1* (downregulated)	hsa-miR-194-5p	CTB-89H12.4;KCNQ1OT1
	hsa-miR-34a-5p	AC004951.6;AC092535.3;KCNQ1OT1;LINC00662;MIR4534;PCBP2-OT1;RP11-693J15.5
	hsa-miR-7-5p	AC005154.6;DLX6-AS1;KCNQ1OT1;LINC01233;LINC01314;MIR4534;OIP5-AS1;OIP5-AS1;RP11-679B19.1;RP1-309I22.2;XIST
*INA* (downregulated)	hsa-miR-212-3p	CASC7;CTB-89H12.4;NEAT1;RP11-26J3.3;XIST;XLOC_006753
	hsa-miR-34a-5p	AC004951.6;AC092535.3;KCNQ1OT1;LINC00662;MIR4534;PCBP2-OT1;RP11-693J15.5
*MLST8* (downregulated)	hsa-miR-7-5p	AC005154.6;DLX6-AS1;KCNQ1OT1;LINC01233;LINC01314;MIR4534;OIP5-AS1;OIP5-AS1;RP11-679B19.1;RP1-309I22.2;XIST
*NDUFV3* (downregulated)	hsa-miR-374a-5p	CTC-444N24.11;CTD-2561J22.5;RP11-613D13.5;TRG-AS1;XIST;XLOC_004545;XLOC_006322;ZNRD1-AS1
	hsa-miR-494-3p	
*NRGN* (downregulated)	hsa-miR-34a-5p	AC004951.6;AC092535.3;KCNQ1OT1;LINC00662;MIR4534;PCBP2-OT1;RP11-693J15.5
*RPH3A* (downregulated)	hsa-miR-136-3p	CTD-2140G10.2;KCNQ1OT1;LL22NC03-2H8.5;RP11-227G15.3;RP11-700J17.2;TTTY15;XLOC_008295
	hsa-miR-221-3p	AC000120.7;CTB-89H12.4;RP11-147L13.8
*RPS27A* (downregulated)	hsa-miR-485-3p	HCG11;LINC00657;RP11-482H16.1;RP11-95O2.1;RP3-445N2.1;XLOC_004244
*SYN1* (downregulated)	hsa-miR-302a-3p	RP11-383H13.1
*TOMM40* (downregulated)	hsa-miR-194-5p	CTB-89H12.4;KCNQ1OT1
*TUBB4B* (downregulated)	hsa-miR-138-5p	KCNQ1OT1;TSIX
	hsa-miR-34a-5p	AC004951.6;AC092535.3;KCNQ1OT1;LINC00662;MIR4534;PCBP2-OT1;RP11-693J15.5
	hsa-miR-494-3p	
*ARFGAP3* (upregulated)	hsa-miR-494-3p	
*CAPN2* (upregulated)	hsa-miR-132-3p	APTR;CASC7;CTB-89H12.4;KCNIP4-IT1;KCNQ1OT1;NEAT1;RP11-26J3.3;XIST;XLOC_006753;YEATS2-AS1
	hsa-miR-494-3p	
	hsa-miR-374a-5p	CTC-444N24.11;CTD-2561J22.5;RP11-613D13.5;TRG-AS1;XIST;XLOC_004545;XLOC_006322;ZNRD1-AS1
*CP* (upregulated)	hsa-miR-34a-5p	AC004951.6;AC092535.3;KCNQ1OT1;LINC00662;MIR4534;PCBP2-OT1;RP11-693J15.5
	hsa-miR-34b-5p	KCNQ1OT1;RP11-458F8.4;TSIX;XIST
	hsa-miR-34c-5p	KCNQ1OT1;LINC01000;MIR4534
	hsa-miR-449a	KCNQ1OT1;MIR4534;XIST
	hsa-miR-449b-5p	KCNQ1OT1;MIR4534;XIST
*DYNLT1* (upregulated)	hsa-miR-34a-5p	AC004951.6;AC092535.3;KCNQ1OT1;LINC00662;MIR4534;PCBP2-OT1;RP11-693J15.5
	hsa-miR-449a	KCNQ1OT1;MIR4534;XIST
*GFAP* (upregulated)	hsa-miR-34a-5p	AC004951.6;AC092535.3;KCNQ1OT1;LINC00662;MIR4534;PCBP2-OT1;RP11-693J15.5
*HSPB8* (upregulated)	hsa-miR-126-5p	AC096772.6;C14orf23;C1QTNF3-AMACR;CH17-262A2.1;DLX6-AS1;FAM201A;KCNQ1OT1;LINC01004;LINC01420;LL22NC03-2H8.5;NEAT1;RP11-177G23.2;RP11-707A18.1;RP11-946L20.2;RP4-740C4.7;TTTY15;XLOC_001417;XLOC_003971;XLOC_005753;XLOC_008295;XLOC_010463
	hsa-miR-485-5p	CTA-342B11.3;GS1-124K5.11;KCNQ1OT1;MIR4534;RP11-266K22.2;RP11-504P24.8;RP11-658F2.8;RP1-309I22.2;RP5-1014D13.2;XLOC_013174;XLOC_013274
	hsa-miR-494-3p	
	hsa-miR-374a-5p	CTC-444N24.11;CTD-2561J22.5;RP11-613D13.5;TRG-AS1;XIST;XLOC_004545;XLOC_006322;ZNRD1-AS1
*IQCK* (upregulated)	hsa-miR-7-5p	AC005154.6;DLX6-AS1;KCNQ1OT1;LINC01233;LINC01314;MIR4534;OIP5-AS1;OIP5-AS1;RP11-679B19.1;RP1-309I22.2;XIST
*LAP3* (upregulated)	hsa-miR-494-3p	
	hsa-miR-449a	KCNQ1OT1;MIR4534;XIST
	hsa-miR-449b-5p	KCNQ1OT1;MIR4534;XIST
*MAN2A1* (upregulated)	hsa-miR-7-5p	AC005154.6;DLX6-AS1;KCNQ1OT1;LINC01233;LINC01314;MIR4534;OIP5-AS1;OIP5-AS1;RP11-679B19.1;RP1-309I22.2;XIST
	hsa-miR-194-5p	CTB-89H12.4;KCNQ1OT1
*MT1M* (upregulated)	hsa-miR-374a-5p	CTC-444N24.11;CTD-2561J22.5;RP11-613D13.5;TRG-AS1;XIST;XLOC_004545;XLOC_006322;ZNRD1-AS1
*NPL* (upregulated)	hsa-miR-374a-5p	CTC-444N24.11;CTD-2561J22.5;RP11-613D13.5;TRG-AS1;XIST;XLOC_004545;XLOC_006322;ZNRD1-AS1
*PLXDC2* (upregulated)	hsa-miR-7-5p	AC005154.6;DLX6-AS1;KCNQ1OT1;LINC01233;LINC01314;MIR4534;OIP5-AS1;OIP5-AS1;RP11-679B19.1;RP1-309I22.2;XIST
	hsa-miR-374a-5p	CTC-444N24.11;CTD-2561J22.5;RP11-613D13.5;TRG-AS1;XIST;XLOC_004545;XLOC_006322;ZNRD1-AS1
*PRDX1* (upregulated)	hsa-miR-485-3p	HCG11;LINC00657;RP11-482H16.1;RP11-95O2.1;RP3-445N2.1;XLOC_004244
	hsa-miR-126-3p	
	hsa-miR-34a-5p	AC004951.6;AC092535.3;KCNQ1OT1;LINC00662;MIR4534;PCBP2-OT1;RP11-693J15.5
*PRDX6* (upregulated)	hsa-miR-34a-5p	AC004951.6;AC092535.3;KCNQ1OT1;LINC00662;MIR4534;PCBP2-OT1;RP11-693J15.5
*RAB10* (upregulated)	hsa-miR-19a-3p	CASC7;FAM201A;H19;KCNA3;KCNQ1OT1;RP11-337C18.8;RP11-523G9.3;SNHG14
	hsa-miR-19b-3p	CASC7;FAM201A;H19;KCNA3;KCNQ1OT1;LINC00094;RP11-337C18.8;SNHG14
*SEPP1* (upregulated)	hsa-miR-218-5p	DYX1C1-CCPG1;INO80B-WBP1;KCNQ1OT1;RP11-166D19.1;RP11-679B19.2;RP4-621B10.8;SNHG23;XLOC_004695;XLOC_010885
	hsa-miR-194-5p	CTB-89H12.4;KCNQ1OT1
	hsa-miR-34b-5p	KCNQ1OT1;RP11-458F8.4;TSIX;XIST
*SERPINA3* (upregulated)	hsa-miR-34a-5p	AC004951.6;AC092535.3;KCNQ1OT1;LINC00662;MIR4534;PCBP2-OT1;RP11-693J15.5
	hsa-miR-34c-5p	KCNQ1OT1;LINC01000;MIR4534
	hsa-miR-448	
*SLC16A9* (upregulated)	hsa-miR-212-3p	CASC7;CTB-89H12.4;NEAT1;RP11-26J3.3;XIST;XLOC_006753
	hsa-miR-374a-5p	CTC-444N24.11;CTD-2561J22.5;RP11-613D13.5;TRG-AS1;XIST;XLOC_004545;XLOC_006322;ZNRD1-AS1
*SRSF6* (upregulated)	hsa-miR-19a-3p	CASC7;FAM201A;H19;KCNA3;KCNQ1OT1;RP11-337C18.8;RP11-523G9.3;SNHG14
	hsa-miR-139-5p	CTC-365E16.1;CTC-444N24.11;HOXA10-HOXA9;KCNQ1OT1;NR2F1-AS1;PWAR6;RMST;RP11-215G15.5;RP13-582O9.5;XLOC_009913
	hsa-miR-494-3p	
	hsa-miR-34a-5p	AC004951.6;AC092535.3;KCNQ1OT1;LINC00662;MIR4534;PCBP2-OT1;RP11-693J15.5
*TAC1* (upregulated)	hsa-miR-212-3p	CASC7;CTB-89H12.4;NEAT1;RP11-26J3.3;XIST;XLOC_006753

**Table 9:** AD-associate miRNAs associated with other neurodegenerative disease-associate DEGs.

**Other neurodegenerative disease-associate DEGs**	**AD-associate miRNAs**	**lncRNAs associated with miRNAs**
*MAP1A*	hsa-miR-24-3p	CTA-292E10.9;CTC-273B12.8;GABPB1-AS1;LINC00662;LOC388692;MIR4534;RP11-54O7.1;XLOC_006242;XLOC_008461;XLOC_011313;
	hsa-miR-497-5p	AC005540.3;C1orf132;C1RL-AS1;FGF14-IT1;GS1-358P8.4;INO80B-WBP1;KCNQ1OT1;LINC00662;MIA-RAB4B;RP11-361F15.2;RP5-991G20.1;RP6-24A23.7;XIST;XLOC_006753;XLOC_008207;XLOC_013174;XLOC_013424
	hsa-miR-15a-5p	AC005540.3;FGF14-IT1;GS1-358P8.4;KCNQ1OT1;LINC00662;MCM3AP-AS1;RP11-361F15.2;RP11-96D1.10;RP3-508I15.20;RP5-991G20.1;RP6-24A23.7;XLOC_003546;XLOC_008207;XLOC_006753;XLOC_013174
	hsa-miR-15b-5p	AC005540.3;FGF14-IT1;GS1-358P8.4;KCNQ1OT1;LINC00662;RP11-361F15.2;RP11-96D1.10;RP3-508I15.20;RP6-24A23.7;XLOC_008207;XLOC_013174
	hsa-miR-16-5p	AC005540.3;FGF14-IT1;GS1-358P8.4;KCNQ1OT1;LINC00662;RP11-359B12.2;RP11-361F15.2;RP3-508I15.20;RP6-24A23.7;XLOC_003546;XLOC_006753;XLOC_008207;XLOC_013174
*MLF2*	hsa-miR-200b-3p	CTC-444N24.11;XIST;XLOC_013174
*NARS*	hsa-miR-15b-5p	AC005540.3;FGF14-IT1;GS1-358P8.4;KCNQ1OT1;LINC00662;RP11-361F15.2;RP11-96D1.10;RP3-508I15.20;RP6-24A23.7;XLOC_008207;XLOC_013174
*ACADM*	hsa-miR-128-3p	
	hsa-miR-376a-5p	KCNQ1OT1;SIK3-IT1
*ACTL6A*	hsa-miR-16-5p	AC005540.3;FGF14-IT1;GS1-358P8.4;KCNQ1OT1;LINC00662;RP11-359B12.2;RP11-361F15.2;RP3-508I15.20;RP6-24A23.7;XLOC_003546;XLOC_006753;XLOC_008207;XLOC_013174
	hsa-miR-18a-5p	AC000403.4;CASC7;CTB-89H12.4;IPW;KCNIP4-IT1;KCNQ1OT1;LINC00506;N4BP2L2-IT2;RP11-10E18.7;RP11-1134I14.8;RP11-147L13.14;RP11-314B1.2;RP11-361F15.2;RP11-707A18.1;RP1-309I22.2;XLOC_003971;XLOC_010463;XLOC_011185;ZNF883;ZNRD1-AS1
	hsa-miR-18b-5p	XIST;XLOC_014102
*AK3*	hsa-miR-16-5p	AC005540.3;FGF14-IT1;GS1-358P8.4;KCNQ1OT1;LINC00662;RP11-359B12.2;RP11-361F15.2;RP3-508I15.20;RP6-24A23.7;XLOC_003546;XLOC_006753;XLOC_008207;XLOC_013174
*AMOTL2*	hsa-miR-107	CASC7;KCNQ1OT1;LINC00662;MIR4534;RP11-361F15.2;RP6-24A23.7;STAG3L5P-PVRIG2P-PILRB;XLOC_006753
	hsa-miR-128-3p	
	hsa-miR-16-5p	AC005540.3;FGF14-IT1;GS1-358P8.4;KCNQ1OT1;LINC00662;RP11-359B12.2;RP11-361F15.2;RP3-508I15.20;RP6-24A23.7;XLOC_003546;XLOC_006753;XLOC_008207;XLOC_013174
*ATG4C*	hsa-miR-128-3p	
	hsa-miR-16-5p	AC005540.3;FGF14-IT1;GS1-358P8.4;KCNQ1OT1;LINC00662;RP11-359B12.2;RP11-361F15.2;RP3-508I15.20;RP6-24A23.7;XLOC_003546;XLOC_006753;XLOC_008207;XLOC_013174
	hsa-miR-200b-3p	CTC-444N24.11;XIST;XLOC_013174
	hsa-miR-31-5p	KCNQ1OT1;TSIX;XLOC_013174
	hsa-miR-376a-5p	KCNQ1OT1;SIK3-IT1
*CAPS2*	hsa-miR-33a-5p	CTC-444N24.11;KCNQ1OT1;MCF2L-AS1
*FUT9*	hsa-miR-33a-5p	CTC-444N24.11;KCNQ1OT1;MCF2L-AS1
*HLA-DMA*	hsa-miR-200b-3p	CTC-444N24.11;XIST;XLOC_013174
*NUP133*	hsa-miR-128-3p	
*PCMTD2*	hsa-miR-200b-3p	CTC-444N24.11;XIST;XLOC_013174
*PLEK*	hsa-miR-107	CASC7;KCNQ1OT1;LINC00662;MIR4534;RP11-361F15.2;RP6-24A23.7;STAG3L5P-PVRIG2P-PILRB;XLOC_006753
*PPM1K*	hsa-miR-107	CASC7;KCNQ1OT1;LINC00662;MIR4534;RP11-361F15.2;RP6-24A23.7;STAG3L5P-PVRIG2P-PILRB;XLOC_006753
	hsa-miR-128-3p	
	hsa-miR-15a-5p	AC005540.3;FGF14-IT1;GS1-358P8.4;KCNQ1OT1;LINC00662;MCM3AP-AS1;RP11-361F15.2;RP11-96D1.10;RP3-508I15.20;RP5-991G20.1;RP6-24A23.7;XLOC_003546;XLOC_008207;XLOC_006753;XLOC_013174
	hsa-miR-15b-5p	AC005540.3;FGF14-IT1;GS1-358P8.4;KCNQ1OT1;LINC00662;RP11-361F15.2;RP11-96D1.10;RP3-508I15.20;RP6-24A23.7;XLOC_008207;XLOC_013174
	hsa-miR-16-5p	AC005540.3;FGF14-IT1;GS1-358P8.4;KCNQ1OT1;LINC00662;RP11-359B12.2;RP11-361F15.2;RP3-508I15.20;RP6-24A23.7;XLOC_003546;XLOC_006753;XLOC_008207;XLOC_013174
	hsa-miR-24-3p	CTA-292E10.9;CTC-273B12.8;GABPB1-AS1;LINC00662;LOC388692;MIR4534;RP11-54O7.1;XLOC_006242;XLOC_008461;XLOC_011313;
	hsa-miR-497-5p	AC005540.3;C1orf132;C1RL-AS1;FGF14-IT1;GS1-358P8.4;INO80B-WBP1;KCNQ1OT1;LINC00662;MIA-RAB4B;RP11-361F15.2;RP5-991G20.1;RP6-24A23.7;XIST;XLOC_006753;XLOC_008207;XLOC_013174;XLOC_013424
*PRPF38B*	hsa-miR-16-5p	AC005540.3;FGF14-IT1;GS1-358P8.4;KCNQ1OT1;LINC00662;RP11-359B12.2;RP11-361F15.2;RP3-508I15.20;RP6-24A23.7;XLOC_003546;XLOC_006753;XLOC_008207;XLOC_013174
*QKI*	hsa-miR-107	CASC7;KCNQ1OT1;LINC00662;MIR4534;RP11-361F15.2;RP6-24A23.7;STAG3L5P-PVRIG2P-PILRB;XLOC_006753
	hsa-miR-128-3p	
	hsa-miR-15a-5p	AC005540.3;FGF14-IT1;GS1-358P8.4;KCNQ1OT1;LINC00662;MCM3AP-AS1;RP11-361F15.2;RP11-96D1.10;RP3-508I15.20;RP5-991G20.1;RP6-24A23.7;XLOC_003546;XLOC_008207;XLOC_006753;XLOC_013174
	hsa-miR-15b-5p	AC005540.3;FGF14-IT1;GS1-358P8.4;KCNQ1OT1;LINC00662;RP11-361F15.2;RP11-96D1.10;RP3-508I15.20;RP6-24A23.7;XLOC_008207;XLOC_013174
	hsa-miR-16-5p	AC005540.3;FGF14-IT1;GS1-358P8.4;KCNQ1OT1;LINC00662;RP11-359B12.2;RP11-361F15.2;RP3-508I15.20;RP6-24A23.7;XLOC_003546;XLOC_006753;XLOC_008207;XLOC_013174
	hsa-miR-24-3p	CTA-292E10.9;CTC-273B12.8;GABPB1-AS1;LINC00662;LOC388692;MIR4534;RP11-54O7.1;XLOC_006242;XLOC_008461;XLOC_011313;
	hsa-miR-29a-3p	AC005154.6;AC006548.28;H19;KCNQ1OT1;LINC00674;MIR4697HG;NEAT1;RP11-314B1.2;RP11-582E3.6;RP4-630A11.3;THUMPD3-AS1;TTTY15;TUG1;XLOC_004366;XLOC_007942;XLOC_008295
	hsa-miR-375	KCNQ1OT1;SNHG14;SNORD116-20
	hsa-miR-497-5p	AC005540.3;C1orf132;C1RL-AS1;FGF14-IT1;GS1-358P8.4;INO80B-WBP1;KCNQ1OT1;LINC00662;MIA-RAB4B;RP11-361F15.2;RP5-991G20.1;RP6-24A23.7;XIST;XLOC_006753;XLOC_008207;XLOC_013174;XLOC_013424
*RNF19A*	hsa-miR-107	CASC7;KCNQ1OT1;LINC00662;MIR4534;RP11-361F15.2;RP6-24A23.7;STAG3L5P-PVRIG2P-PILRB;XLOC_006753
	hsa-miR-29a-3p	AC005154.6;AC006548.28;H19;KCNQ1OT1;LINC00674;MIR4697HG;NEAT1;RP11-314B1.2;RP11-582E3.6;RP4-630A11.3;THUMPD3-AS1;TTTY15;TUG1;XLOC_004366;XLOC_007942;XLOC_008295
*SLC44A1*	hsa-miR-24-3p	CTA-292E10.9;CTC-273B12.8;GABPB1-AS1;LINC00662;LOC388692;MIR4534;RP11-54O7.1;XLOC_006242;XLOC_008461;XLOC_011313
*SPP1*	hsa-miR-16-5p	AC005540.3;FGF14-IT1;GS1-358P8.4;KCNQ1OT1;LINC00662;RP11-359B12.2;RP11-361F15.2;RP3-508I15.20;RP6-24A23.7;XLOC_003546;XLOC_006753;XLOC_008207;XLOC_013174
*STARD7*	hsa-miR-31-5p	KCNQ1OT1;TSIX;XLOC_013174
	hsa-miR-433-3p	
*STOM*	hsa-miR-107	CASC7;KCNQ1OT1;LINC00662;MIR4534;RP11-361F15.2;RP6-24A23.7;STAG3L5P-PVRIG2P-PILRB;XLOC_006753
	hsa-miR-128-3p	
	hsa-miR-15a-5p	AC005540.3;FGF14-IT1;GS1-358P8.4;KCNQ1OT1;LINC00662;MCM3AP-AS1;RP11-361F15.2;RP11-96D1.10;RP3-508I15.20;RP5-991G20.1;RP6-24A23.7;XLOC_003546;XLOC_008207;XLOC_006753;XLOC_013174
	hsa-miR-15b-5p	AC005540.3;FGF14-IT1;GS1-358P8.4;KCNQ1OT1;LINC00662;RP11-361F15.2;RP11-96D1.10;RP3-508I15.20;RP6-24A23.7;XLOC_008207;XLOC_013174
	hsa-miR-16-5p	AC005540.3;FGF14-IT1;GS1-358P8.4;KCNQ1OT1;LINC00662;RP11-359B12.2;RP11-361F15.2;RP3-508I15.20;RP6-24A23.7;XLOC_003546;XLOC_006753;XLOC_008207;XLOC_013174
	hsa-miR-497-5p	AC005540.3;C1orf132;C1RL-AS1;FGF14-IT1;GS1-358P8.4;INO80B-WBP1;KCNQ1OT1;LINC00662;MIA-RAB4B;RP11-361F15.2;RP5-991G20.1;RP6-24A23.7;XIST;XLOC_006753;XLOC_008207;XLOC_013174;XLOC_013424
*SUMF1*	hsa-miR-128-3p	
	hsa-miR-16-5p	AC005540.3;FGF14-IT1;GS1-358P8.4;KCNQ1OT1;LINC00662;RP11-359B12.2;RP11-361F15.2;RP3-508I15.20;RP6-24A23.7;XLOC_003546;XLOC_006753;XLOC_008207;XLOC_013174
*TRIM22*	hsa-miR-107	CASC7;KCNQ1OT1;LINC00662;MIR4534;RP11-361F15.2;RP6-24A23.7;STAG3L5P-PVRIG2P-PILRB;XLOC_006753
	hsa-miR-31-5p	KCNQ1OT1;TSIX;XLOC_013174
*TSPAN6*	hsa-miR-107	CASC7;KCNQ1OT1;LINC00662;MIR4534;RP11-361F15.2;RP6-24A23.7;STAG3L5P-PVRIG2P-PILRB;XLOC_006753
	hsa-miR-16-5p	AC005540.3;FGF14-IT1;GS1-358P8.4;KCNQ1OT1;LINC00662;RP11-359B12.2;RP11-361F15.2;RP3-508I15.20;RP6-24A23.7;XLOC_003546;XLOC_006753;XLOC_008207;XLOC_013174
*VPS13C*	hsa-miR-107	CASC7;KCNQ1OT1;LINC00662;MIR4534;RP11-361F15.2;RP6-24A23.7;STAG3L5P-PVRIG2P-PILRB;XLOC_006753
	hsa-miR-16-5p	AC005540.3;FGF14-IT1;GS1-358P8.4;KCNQ1OT1;LINC00662;RP11-359B12.2;RP11-361F15.2;RP3-508I15.20;RP6-24A23.7;XLOC_003546;XLOC_006753;XLOC_008207;XLOC_013174
	hsa-miR-186-5p	CTA-292E10.9;CTB-89H12.4;CTC-428G20.3;MAGI2-AS3;MIR4534;NEAT1;OIP5-AS1;RP11-61A14.2;RP1-309I22.2;XIST
	hsa-miR-29a-3p	AC005154.6;AC006548.28;H19;KCNQ1OT1;LINC00674;MIR4697HG;NEAT1;RP11-314B1.2;RP11-582E3.6;RP4-630A11.3;THUMPD3-AS1;TTTY15;TUG1;XLOC_004366;XLOC_007942;XLOC_008295

### Analysis of miRNA Expression in AD by High-Throughput Data

To study the predicted expression of the AD-associated miRNAs further, a GEO dataset (GSE16759) studying miRNA was analyzed. This revealed 870 differentially expressed miRNAs. Detailed information on these miRNAs is shown in Table S7. Then, we analyzed the miRNAs common to both GSE16759 and our AD-associated miRNAs, and detected 47 of our AD-associated miRNAs in the GEO data. The top 12 significant miRNAs common to both GSE16759 and our AD-associated miRNAs are listed in Table 10.

**Table 10:** The top significant common genes in GSE16759 and our AD-associated miRNA.

**ID**	**adj.*P*-Value**	***P*-Value**	**logFC**
hsa-miR-424	0.0889	0.0011322	−3.6568626
hsa-miR-376a	0.1033	0.0068405	−3.47133
hsa-miR-186	0.1028	0.0025921	−2.5463854
hsa-miR-148b	0.1028	0.0061566	−2.2428498
hsa-miR-101	0.0889	0.000485	−1.8992436
hsa-miR-340	0.3401	0.1038393	−1.7586512
hsa-miR-29b	0.0889	0.0014182	−1.6383336
hsa-miR-15a	0.1028	0.0040077	−1.524008
hsa-miR-137	0.1262	0.0127089	−1.4923006
hsa-miR-130a	0.1028	0.0066478	−1.4733294
hsa-miR-29c	0.1033	0.0069833	−1.4723696
hsa-miR-27a	0.1375	0.0155005	−1.011425

### The Transcription Factors Associated With the AD-Associated/Other Neurodegenerative Disease-Associated DEGs and miRNAs

By analyzing TF–gene regulation, we found 442 gene-associated TFs (gTFs) associated with 55 AD-associated DEGs (Table S8), and 400 gTFs associated with 42 other neurodegenerative disease-associated DEGs (Table S9).

By studying the regulatory relationships between TFs and miRNAs, we obtained 253 miRNA-associated TFs (mTFs) associated with 50 AD-associated miRNAs (Table S10), and 118 mTFs associated with 22 other neurodegenerative disease-associated miRNAs whose target genes were identified as AD-associated DEGs in our study (Table S11).

### mTF–miRNA–gene–gTF Regulatory Network

A regulatory network was constructed to study the regulatory interactions further, containing the AD-associated DEGs, the TFs associated with these genes (gTFs), the AD-associated miRNAs associated with these genes, the other neurodegenerative disease-associated miRNAs targeting these DEGs, and the TFs related to these miRNAs (mTFs). To study the other neurodegenerative disease-associated DEGs further, the TFs associated with these genes (gTFs), the AD-associated miRNAs targeting these genes, and the TFs related to these miRNAs (mTFs) were also analyzed.

This showed that *NFASC* and *ADAP1* are regulated by the most gTFs, 114 and 111, respectively. *NFASC* is involved in multiple sclerosis (Kawamura, 2014), and *ADAP1* is involved in AD (Stricker and Reiser, 2014). We also found gTFs for the hub genes Glyceraldehyde-3-phosphate dehydrogenase (*GAPDH*), ribosomal protein S27a *(RPS27A*), Glial fibrillary acidic protein (*GFAP)*, Beta-2 microglobulin (*B2M)*, Clusterin (*CLU)*, Eukaryotic elongation factor 2 (*EEF2)*, Gap junction protein alpha 1 (*GJA1)*, and Ceruloplasmin (*CP*).

*RAB10* and *TUBB4B* are regulated by the most miRNAs, 25 and 16, respectively. Both genes have been reported to be associated with AD (Olah et al., 2011; Martins-de-Souza et al., 2012).

**Figure 6:**
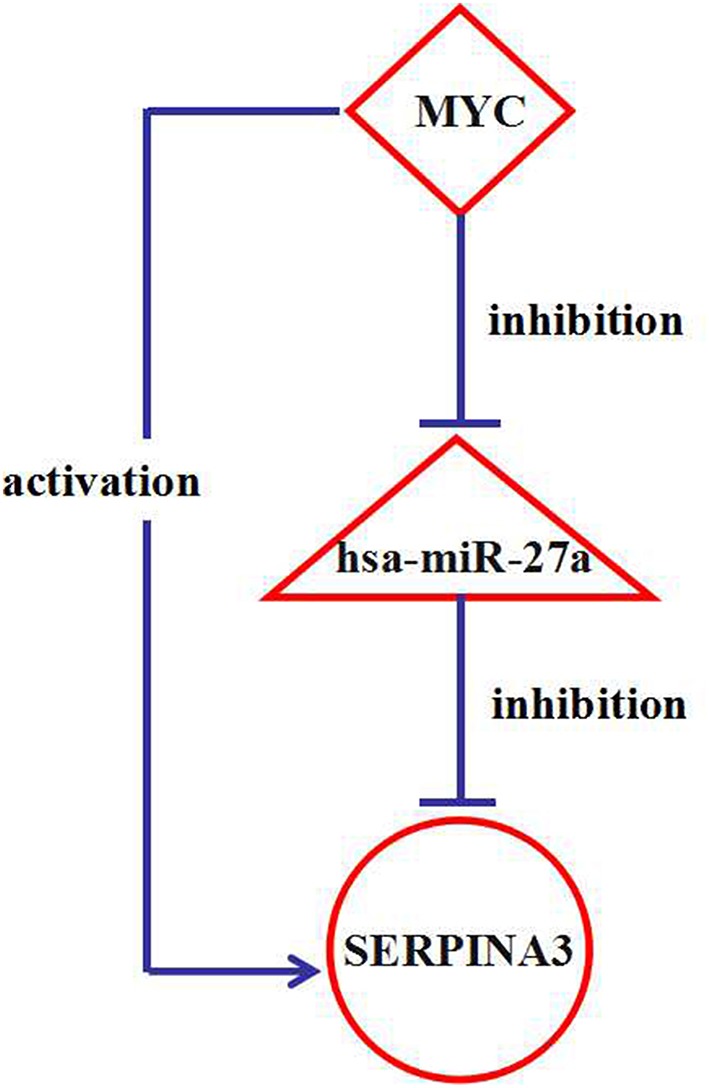
The interaction network among a FFL nodes. The triangle-shaped node represents miRNAs, ellipse-shaped node represents genes, and diamond-shaped node represents transcription factors (TFs).

Analysis of the regulatory network identified the presence of 131 interesting feed-forward loops (FFLs) (Table S12), in which a TF controls a miRNA and together they coregulate a target gene. These 131 FFLs involved 22 DEGs (20 AD-associated DEGs and two other neurodegenerative disease-associated DEGs), 31 miRNAs (26 AD-associated miRNAs and five other neurodegenerative disease-associated miRNAs), and 28 TFs. It was interesting that an FFL was identified between the gene *SERPINA3*, hsa-miR-27a, and the TF MYC, shown in Figure 6. Interestingly, our study found that two other neurodegenerative disease-associated DEGs are involved in such FFLs between the gene *STARD7*, hsa-miR-433, and the TF SMAD3, the gene *STARD7*, hsa-miR-31, and the TF SMAD3, the gene *TRIM22*, hsa-miR-31, and the TF ELK1, the gene *TRIM22*, hsa-miR-31, and the TF SMAD3, and the gene *TRIM22*, hsa-miR-31, and the TF SOX4. Hsa-miR-433 and hsa-miR-31 have already been reported to be associated with AD. Therefore, the associations between AD and the genes *STARD7* and *TRIM22* were studied further.

### Verification of FFL Between the Gene SERPINA3, hsa-miR-27a, and TF MYC

GEO dataset (GSE16759) studying mRNA and miRNA was analyzed. This result revealed that SERPINA3 and MYC are upregulated, and hsa-miR-27a is downregulated in AD patients compared with controls. Detailed information is shown in Table 11.

**Table 11:** The expression the gene SERPINA3, hsa-miR-27a, and TF MYC from FFL in different datasets.

**Gene/miRNA symbol**	**GSE16759**			**GSE97760(mRNA)/GSE46579(miRNA)**		
	**adj.*P*-Value**	***P*-Value**	**logFC**	**adj.*P*-Value**	***P*-Value**	**logFC**
*SERPINA3*	0.999	0.6698836	0.41191464	0.000878	0.0000481	2.3410491
*MYC*	0.988	0.2150454	0.43630728	0.541	0.323	−0.3806443
hsa-miR-27a	0.1375	0.0155005	−1.011425	0.42917903	0.576982734	−0.187196408

To further verify the results, GSE46579 dataset studying miRNA expression in AD patients and controls blood was analyzed. Hsa-miR-27a was shown downregulated in AD patients blood. GSE97760 dataset studying mRNA expression was analyzed. The expression of SERPINA3 was shown upregulated, however MYC was shown downregulated. Detailed information is shown in Table 11.

### SNP Analysis of the AD-Associated DEGs

SNPs corresponding to the AD-associated DEGs were obtained from the MirSNP online database. This showed that 1051 miRNAs were related to these SNPs, of which 79 miRNAs were AD associated. The results showed that 173 SNPs were related to these 79 miRNAs, and these 173 SNPs were associated with 40 AD-associated DEGs identified in our study. The chromosome loci information of these 173 SNPs is shown in Table S13.

## Discussion

In the past decades, research on the progression of AD has been productive, however identification of more potential genes and pathways in the pathogenesis of AD is needed. Therefore, large sample studies are essential for studying the effects of genes on the development of AD, and meta-analysis allows new biological insights.

In this study, we obtained DEGs by meta-analysis merging several AD-related microarray gene expression studies. This resulted in eight hubs with high degree and closeness centrality values, which are *GAPDH, RPS27A, GFAP, B2M, CLU, EEF2, GJA1*, and *CP*.

GAPDH co-localizes with most neurofibrillary tangles in the AD brain, and co-immunoprecipitates with abnormal tau antibodies in AD (Wang et al., 2005). The expression level of *GAPDH* in blood samples from familial AD patients is decreased compared with healthy controls (El Kadmiri et al., 2014).

RPS27A, a component of the 40S subunit of the ribosome, are associated with AD (Soler-Lopez et al., 2011).

GFAP, an astrocyte-specific intermediate filament, is significantly increased in AD mouse models compared with wildtype mice (Kamphuis et al., 2012).

B2M is the light chain of the first major histocompatibility (MHC) antigen. Increased plasma B2M results in deposition of amyloid fibrils, which is associated with over 20 degenerative diseases, including AD (Kardos et al., 2004).

CLU protein, an apolipoprotein, is responsible for clearing amyloid peptide and has neuroprotective effects for AD (Karch and Goate, 2015).

EEF2 is significantly decreased in AD compared with controls (Li et al., 2005).

GJA1, also known as connexin 43, shows upregulated mRNA and protein levels in AD (Ren et al., 2018). Specific deletion of astroglial connexin 43 in AD mice improved cognitive dysfunction (Ren et al., 2018).

CP, a ferrous oxidase enzyme, plays an important role in regulating iron metabolism and redox reactions. *CP* expression was significantly downregulated in the hippocampus region of AD patients, resulting in memory impairment and increased iron accumulation (Zhao et al., 2018).

However, the role of these genes in AD is not very clear. The importance of these genes requires further study in AD.

Studies also have shown that there is a difference between different sexes in neuroanatomy and function, so gender differences may be of great significance in the treatment of AD (Moradifard et al., 2018). More and more attention has been paid to the gender differences in AD prevalence. Some evidence showed that the risk of AD due to APOE ε4 allele is different in two sexes: female carriers are at higher risk of AD than male carriers (Nyarko et al., 2018). Based on our results, there are also a number of genes which specifically expressed in male and female. Such as CHC22 protein encoded by *CLTCL1* gene was strongly suggested to play important function in affecting neuronal progenitor cells or immature neurons, and *CLTCL1* was significantly upregulated in the development of human brain, especially cerebral cortex (Nahorski et al., 2015). LPIN1 plays a role in abdominal obesity, insulin sensitivity, and hypertriglyceridemia and it is associated to blood pressure regulation, especially in men (Ong et al., 2008). FOLR1 was reported to be overexpressed in ovarian cancer (Lin et al., 2013), and its expression could be regulated by both female sex hormones and retinoic acid (Kelemen et al., 2014). ELAVL2 might be critical for normal neuronal and synaptic function in the brain by regulating important genetic pathways participated in human neurodevelopment (Berto et al., 2016).

Analysis of FFL identified the bioregulatory relationship between the gene SERPINA3, hsa-miR-27a, and the TF MYC. TransmiR information revealed that hsa-miR-27a is repressed by the TF MYC. By combining the results of the DIANA-LncBase and TRANSFAC databases, both MYC and hsa-miR-27a were found to regulate the target gene *SERPINA3*. The expression of hsa-miR-27a is downregulated in AD patients compared with controls (Nunez-Iglesias et al., 2010), this result is also verified in our study and GSE46579 dataset (Table 11). MYC is also physiologically relevant and is increased in vulnerable neurons in patients with AD (Ferrer et al., 2001). This suggests that this TF may be highly expressed in the brain tissues of AD patients, indicating that MYC downregulates hsa-miR-27a. SERPINA3 is also involved in AD (Guan et al., 2012) and was detected to be upregulated in our study. Therefore, this validates our findings in AD. It also verifies that MYC and *SERPINA3* have an activation relationship. This activation relationship is also verified in the GEO dataset GSE16759. However, in GSE97760, the result is different to ours and GSE16759, which may be due to different samples (The samples in GSE16759 were the tissues, however the samples in GSE97760 were blood).

There were 173 SNPs identified as being associated with 40 AD-associated DEGs, which were in turn regulated by AD-associated miRNAs. This enhances the relevance of these 173 SNPs in AD (Table S13). The function of these 173 SNPs was further analyzed using the SNP annotation tool SNPnexus (http://snp-nexus.org/index.html) (Dayem Ullah et al., 2018). Several SNPs related to hsa-miR-27a were identified. Among them were SNPs rs76463641 and rs79339279, located at the *BRSK2* and *GNB5* loci, respectively, which are both already known to be AD-associated genes (Katsumata et al., 2019). Therefore, hsa-miR-27a may be an important AD epigenetic biomarker in our study.

Furthermore, our study identified several SNPs associated with five other neurodegenerative disease-associated miRNAs involved in FFL of the regulatory network. Interestingly, SNPs associated with hsa-miR-34c, hsa-miR-212, hsa-miR-34a, and hsa-miR-7 are located at known AD-associated gene loci. Thus, these four miRNAs may be associated with AD, although this requires further study for confirmation.

Although this study is strict, it has some limitations. As the power of gene analysis is affected by the sample size, especially the number of cases, the current study may not have the strongest power. In addition, not all brain regions were studied. The other limitation is that only one dataset on the HIP brain region was selected to validate the microarray results, as RNA-Seq data for the other brain regions we studied (EC and MTG) in AD is lacking. So, further study is required, possibly with larger sample sizes, more brain regions. Considering the latent effects of the identified biomolecules in the pathogenesis of AD, experimental studies should be conducted to determine the possible roles of these molecules, but are lacking. In addition, the FFL between the gene *SERPINA3*, hsa-miR-27a and the TF MYC possibly provides new candidates for treatment of AD, so further experimental verification is needed.

## Conclusion

In this study, based on high degree and closeness centrality values in the gene expression network, eight hub genes were identified, all of which have been reported to be associated with AD. By analyzing the mTF-miRNA-gene-gTF regulatory network, 131 FFLs were identified, in which an important FFL between the gene *SERPINA3*, hsa-miR-27a, and the TF MYC was identified. Further study on the lncRNA-mediated regulatory network suggested that these lncRNAs may be significant in AD, and these have not been found in previous studies. Moreover, 173 important SNPs were identified by SNP analysis, which may be helpful for predicting AD at an earlier stage.

## Data Availability

All the data supporting the results of this study are included in the manuscript and the related Supplementary Documents.

## Author Contributions

LS and HW designed the study. LS, SC, CZ, HW, and XS performed the data analysis. LS wrote the manuscript. HW supervised this work. All authors read and approved the final manuscript.

### Conflict of Interest Statement

CZ was employed by company Shenzhen RealOmics (Biotech) Co., Ltd. The remaining authors declare that the research was conducted in the absence of any commercial or financial relationships that could be construed as a potential conflict of interest.

## Supplementary Material

Additional Figures All cassette figures of the expression data after standardization.

Table S1 Detailed descriptions of the samples including the brain regions, sex and mean age.

Table S2 Detailed information on the RNA-Seq samples collected from GEO data GSE67333.

Table S3 Results of sub-meta-analysis of EC brain regions on males and females.

Table S4 Results of sub-meta-analysis of HIP brain regions on males and females.

Table S5 Results of sub-meta-analysis of MTG brain regions on males and females.

Table S6 Detailed information on the DEGs in RNA-Seq data set GSE67333.

Table S7 Detailed information on the differentially expressed miRNAs from GEO data GSE16759.

Table S8 gTFs associated with AD-associated DEGs.

Table S9 gTFs associated with other neurodegenerative disease-associated DEGs.

Table S10 mTFs associated with AD-associated miRNAs.

Table S11 mTFs associated with other neurodegenerative disease-associated miRNAs.

Table S12 Feed-forward loop from the mTF–miRNA–gene–gTF regulatory networks.

Table S13 Important SNPs in AD and their related AD-associated miRNAs and genes.

## Abbreviations

DEGs: differentially expressed genes
gTF: transcription factor associated with gene
lncRNA: long non-coding RNA
miRNA: microRNA
mTF: transcription factor associated with miRNA
AD: Alzheimer's disease
SNP: single nucleotide polymorphism
TF: transcription factor
PPI: protein–protein interaction
FFL: feed-forward loop.
