# Supplementary material for: Meta-Analysis of Gene Expression and Identification of Biological Regulatory Mechanisms in Alzheimer's Disease
Source: Front Neurosci. 2019 Jul 3;13:633. doi: 10.3389/fnins.2019.00633 (PMC6616202; doi:10.3389/fnins.2019.00633)
Supplement: Additional Figures — All cassette figures of the expression data after standardization. [file Data_Sheet_1.docx]

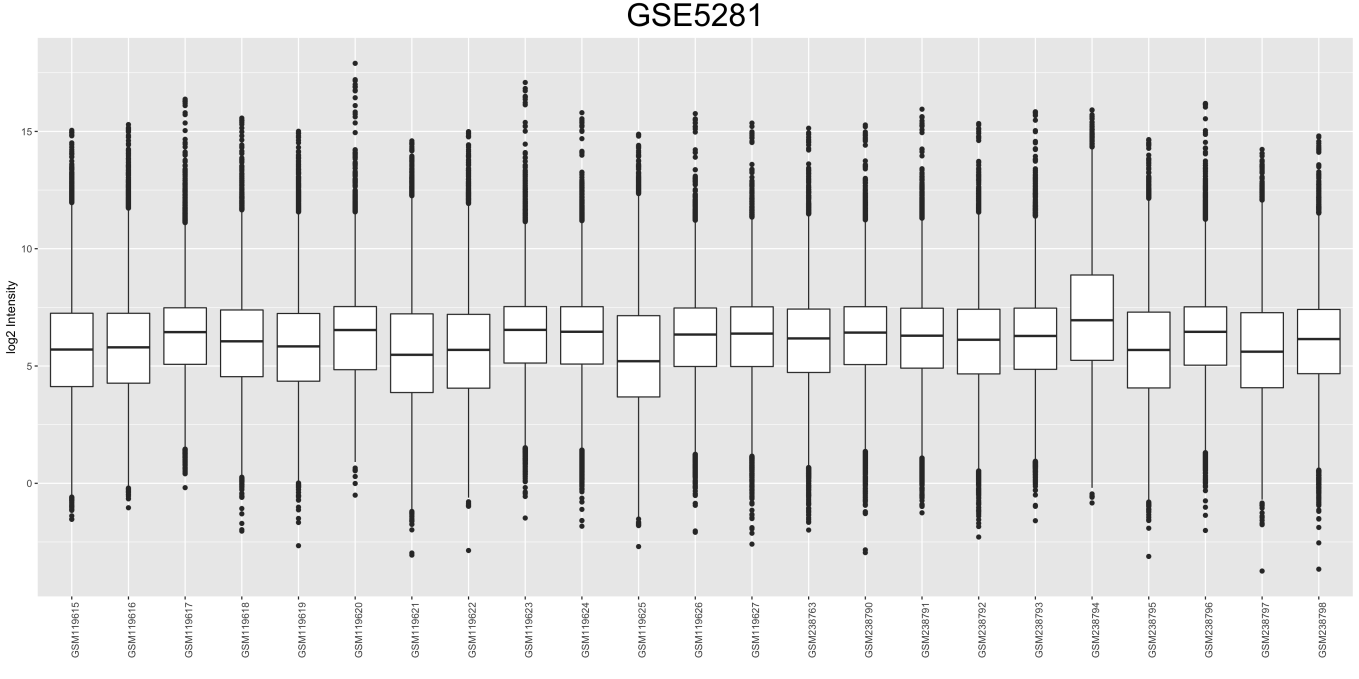


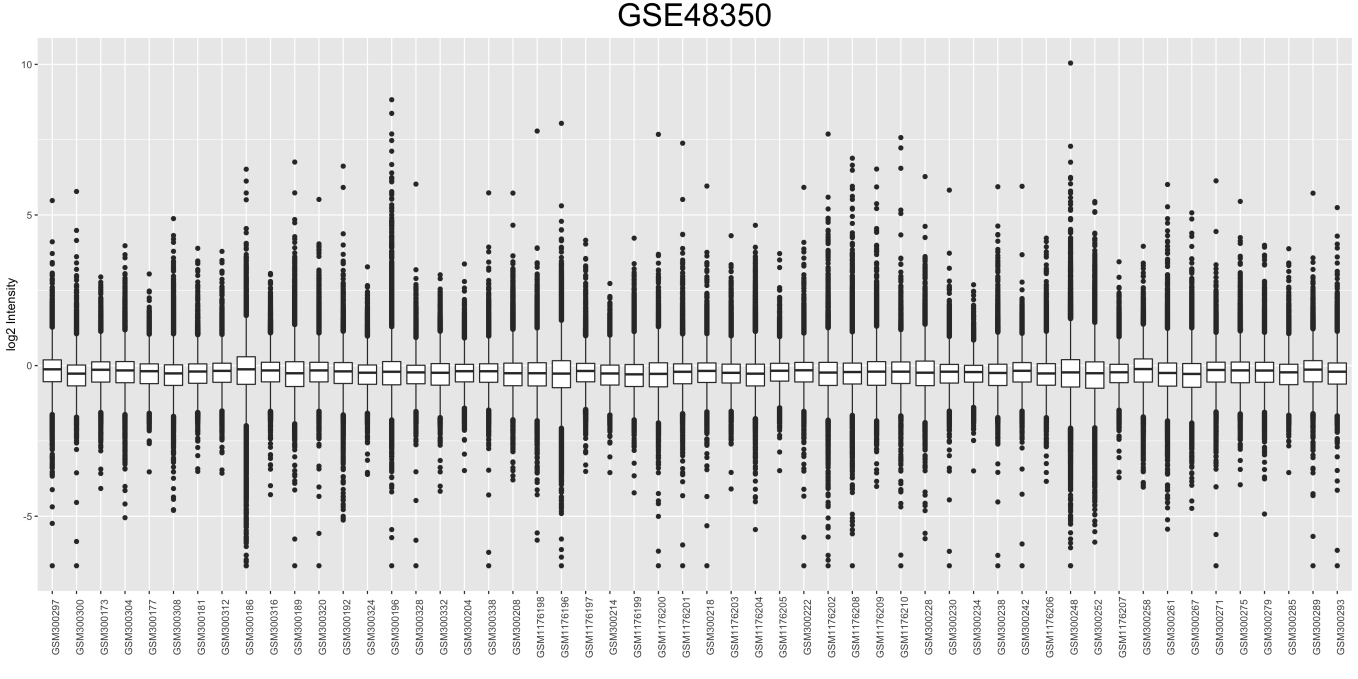


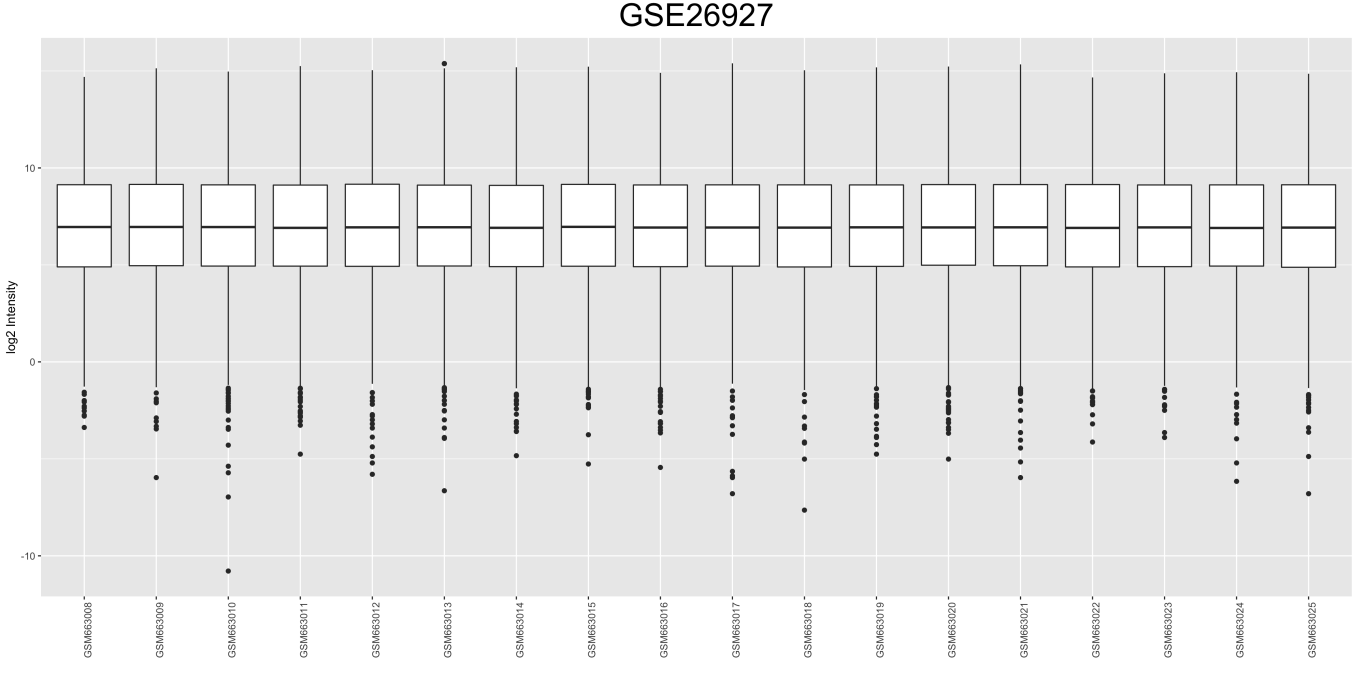
**Figure 1 Cassette figures of the expression data from brain regions entorhinal cortex (EC) after standardization**


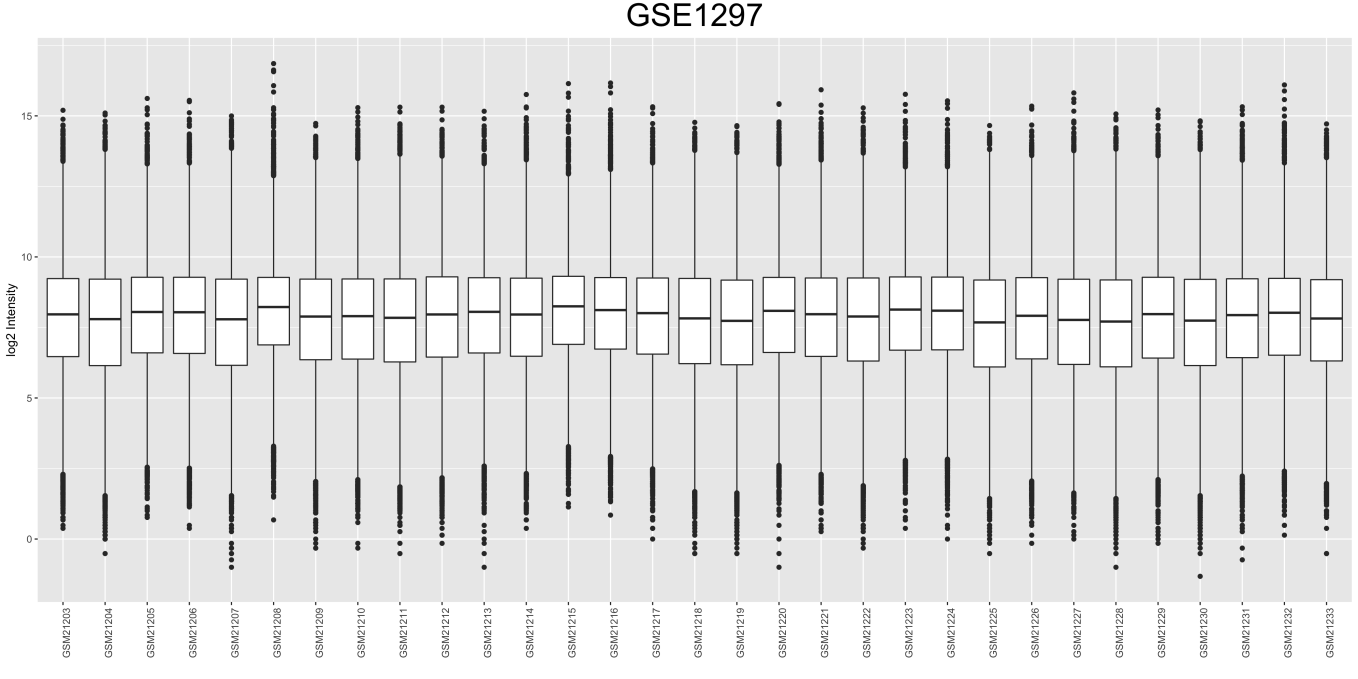


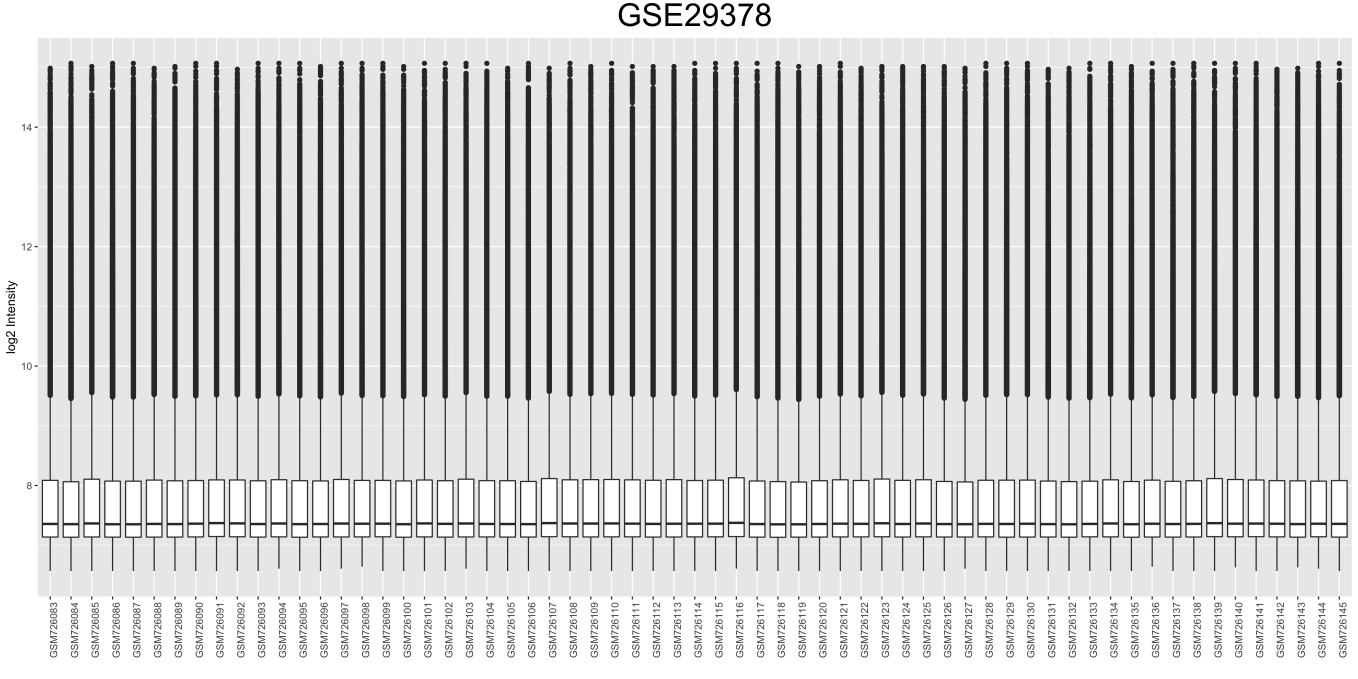

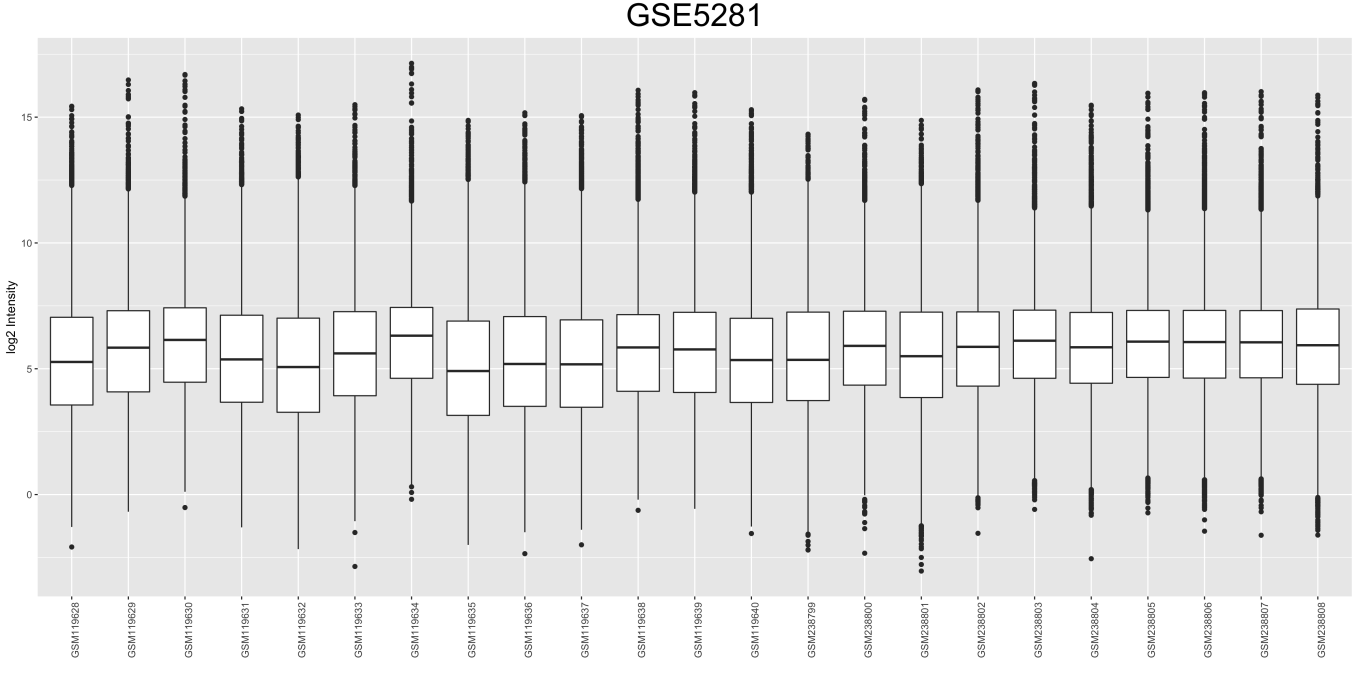


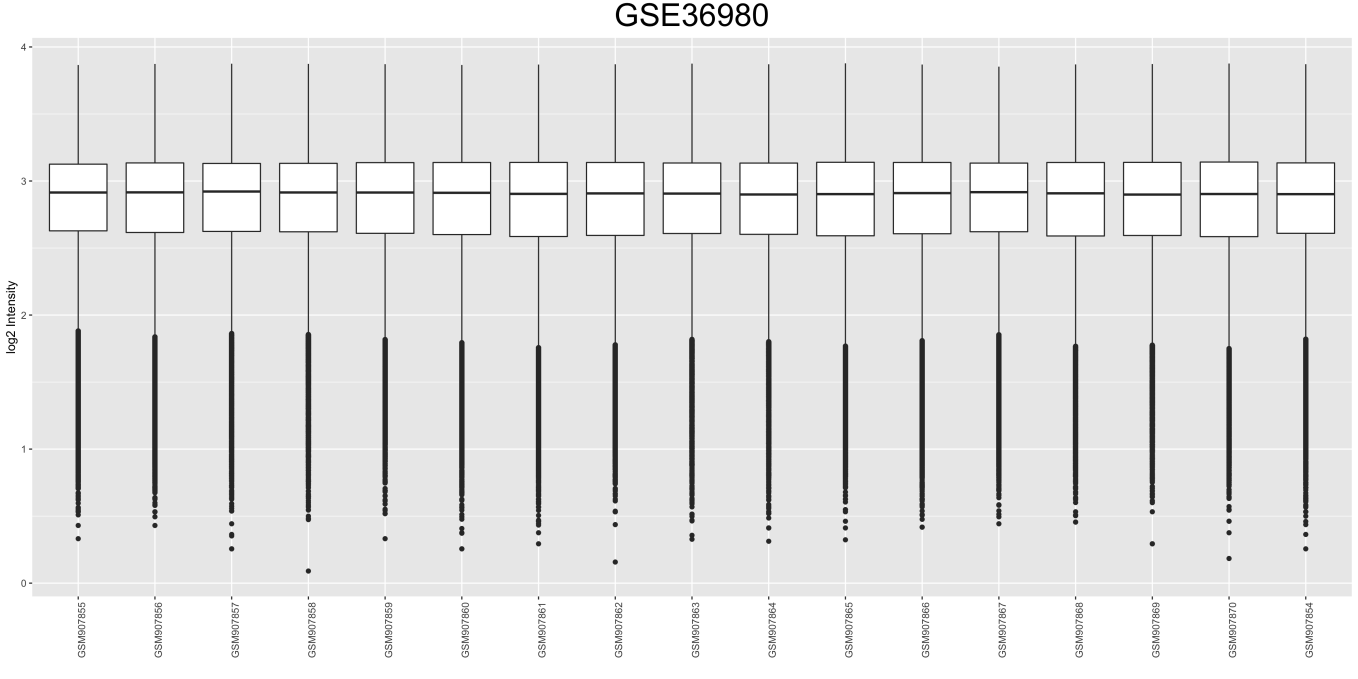


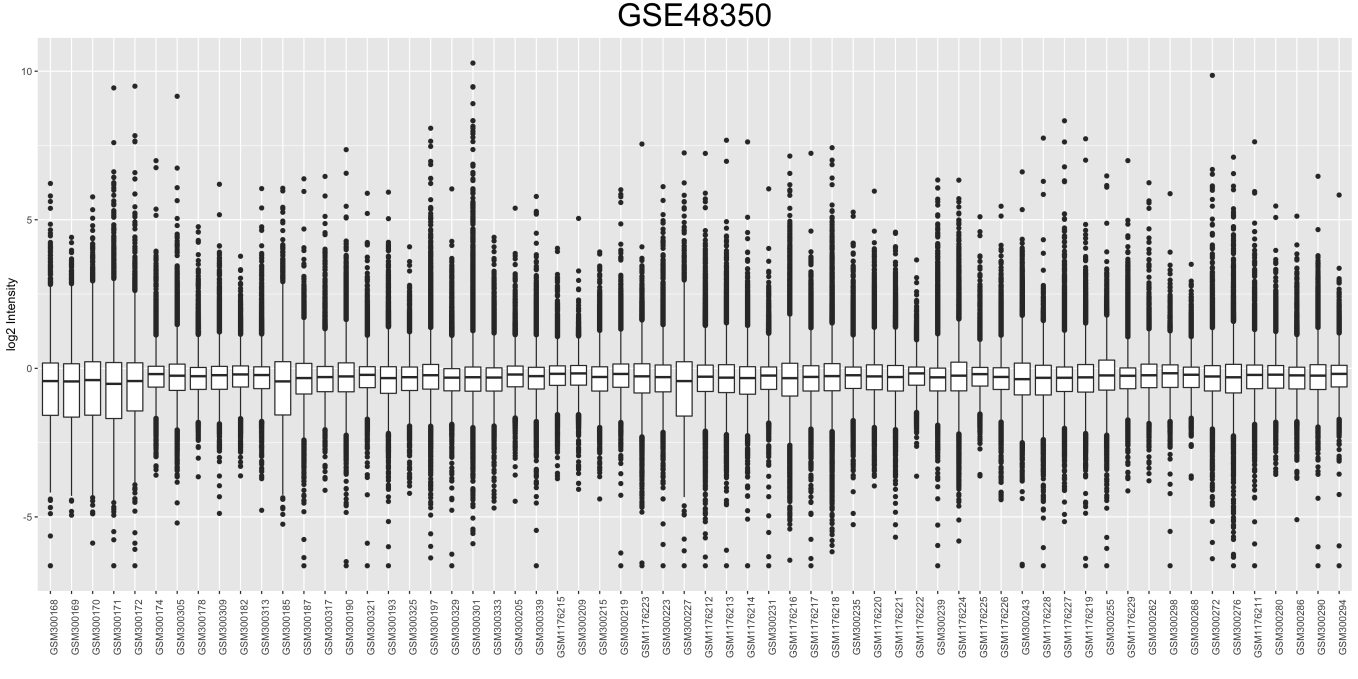


**Figure 2 Cassette figures of the expression data from brain regions hippocampus (HIP) after standardization**


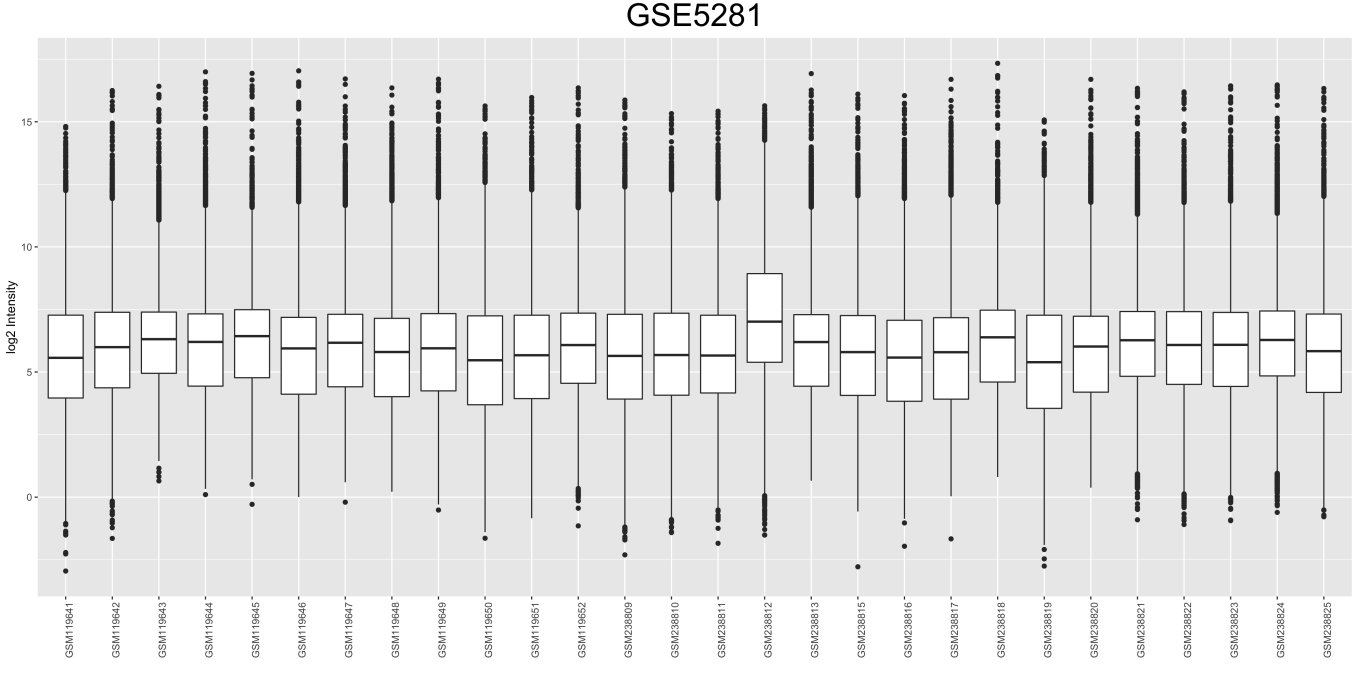


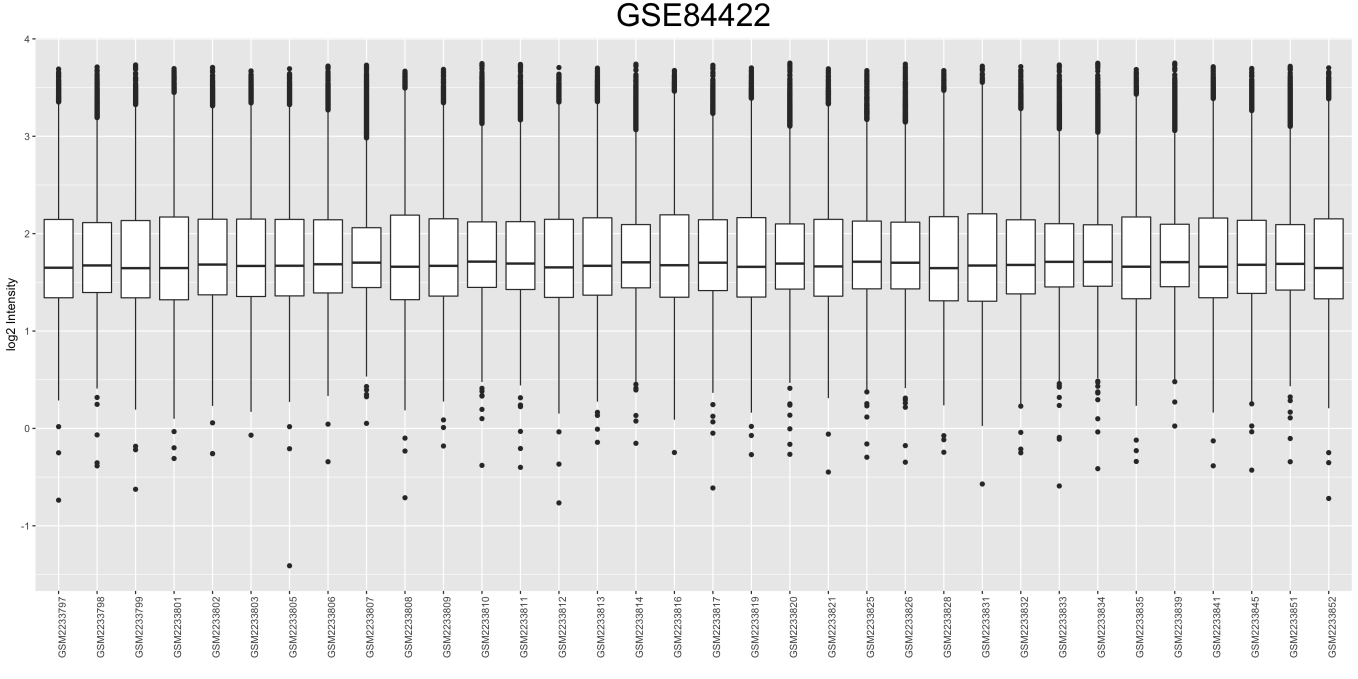


**Figure 3 Cassette figures of the expression data from brain regions medial temporal gyrus（MTG）after standardization**
