# Supplementary material for: Meta-Analysis of Gene Expression and Identification of Biological Regulatory Mechanisms in Alzheimer's Disease
Source: Front Neurosci. 2019 Jul 3;13:633. doi: 10.3389/fnins.2019.00633 (PMC6616202; doi:10.3389/fnins.2019.00633)
Supplement: Table S1 — Detailed descriptions of the samples including the brain regions, sex and mean age. [file Table_1.doc]

| GSE ID | Sex（control/case） | Brain region | Sample collection(country/city) | Mean age  (control/AD) |
| --- | --- | --- | --- | --- |
| GSE48350 | 17M,22F/7M,8F | Entorhinal Cortex (EC) | USA//Irvine | 55.3/86.5 |
| GSE5281 | 10M,3F/ 4M, 6F | Entorhinal Cortex (EC) | USA/Phoenix | 80.3/85.6 |
| GSE5281 | 9M,4F/ 6M, 4F | Hippocampus (HIP) | USA/Phoenix | 79.6/77.8 |
| GSE36980 | 5M,5F/ 3M, 4F | Hippocampus (HIP) | Japan/Fukuoka | 77/92.9 |
| GSE1297 | 5M,5F/ 3M, 4F | Hippocampus (HIP) | USA/Lexington | 85.3/86.3 |
| GSE29378 | 22M,10F/ 16M, 15F | Hippocampus (HIP) | USA/Lexington | 81.7/76.6 |
| GSE48350 | 23M,20F/ 9M, 10F | Hippocampus (HIP) | USA//Irvine | 62.5/83.1 |
| GSE5281 | 8M,4F/ 10M, 6F | Medial temporal gyrus（MTG） | USA/Phoenix | 80.1/79.1 |
| GSE84422 | 6M, 8F/ 8M,12F | Medial temporal gyrus（MTG） | USA/New York | 81.3/86.4 |
