# Supplementary material for: Meta-Analysis of Gene Expression and Identification of Biological Regulatory Mechanisms in Alzheimer's Disease
Source: Front Neurosci. 2019 Jul 3;13:633. doi: 10.3389/fnins.2019.00633 (PMC6616202; doi:10.3389/fnins.2019.00633)
Supplement: Table S2 — Detailed information on the RNA-Seq samples collected from GEO data GSE67333. [file Table_2.doc]

| GSE ID | Sex（control/case） | Brain region | Sample collection(country/city) | Mean age  (control/AD) |
| --- | --- | --- | --- | --- |
| GSE67333 | 2M,2F/1M,3F | Hippocampus (HIP) | USA/Branner Sun Health Research Institute | 83.8/83.5 |
